# Transcriptome Response and Spatial Pattern of Gene Expression in the Primate Subventricular Zone Neurogenic Niche After Cerebral Ischemia

**DOI:** 10.3389/fcell.2020.584314

**Published:** 2020-12-03

**Authors:** Monika C. Chongtham, Haifang Wang, Christina Thaller, Nai-Hua Hsiao, Ivan H. Vachkov, Stoyan P. Pavlov, Lorenz H. Williamson, Tetsumori Yamashima, Anastassia Stoykova, Jun Yan, Gregor Eichele, Anton B. Tonchev

**Affiliations:** ^1^Department of Genes and Behavior, Max Planck Institute for Biophysical Chemistry, Göttingen, Germany; ^2^Institute of Neuroscience, State Key Laboratory of Neuroscience, CAS Center for Excellence in Brain Science and Intelligence Technology, Shanghai Institutes for Biological Sciences, Chinese Academy of Sciences, Shanghai, China; ^3^Department of Anatomy and Cell Biology, Faculty of Medicine, Medical University, Varna, Bulgaria; ^4^Department of Stem Cell Biology and Advanced Computational Bioimaging, Research Institute, Medical University, Varna, Bulgaria; ^5^Department of Psychiatry and Behavioral Science, Kanazawa University Graduate School of Medical Sciences, Kanazawa, Japan

**Keywords:** neural stem cell, global ischemia, non-human primate, subventricular zone, cell marker

## Abstract

The main stem cell niche for neurogenesis in the adult mammalian brain is the subventricular zone (SVZ) that extends along the cerebral lateral ventricles. We aimed at characterizing the initial molecular responses of the macaque monkey SVZ to transient, global cerebral ischemia. We microdissected tissue lining the anterior horn of the lateral ventricle (SVZa) from 7 day post-ischemic and sham-operated monkeys. Transcriptomics shows that in ischemic SVZa, 541 genes were upregulated and 488 genes were down-regulated. The transcription data encompassing the upregulated genes revealed a profile typical for quiescent stem cells and astrocytes. In the primate brain the SVZ is morphologically subdivided in distinct and separate ependymal and subependymal regions. The subependymal contains predominantly neural stem cells (NSC) and differentiated progenitors. To determine in which SVZa region ischemia had evoked transcriptional upregulation, sections through control and ischemic SVZa were analyzed by high-throughput *in situ* hybridization for a total of 150 upregulated genes shown in the *www.monkey-niche.org* image database. The majority of the differentially expressed genes mapped to the subependymal layers on the striatal or callosal aspect of the SVZa. Moreover, a substantial number of upregulated genes was expressed in the ependymal layer, implicating a contribution of the ependyma to stem cell biology. The transcriptome analysis yielded several novel gene markers for primate SVZa including the apelin receptor that is strongly expressed in the primate SVZa niche upon ischemic insult.

## Introduction

Neurogenesis in the adult mammalian brain takes place in two niches, the subventricular zone (SVZa) lining the anterior horn of the forebrain lateral ventricle and the subgranular zone (SGZ) of the dentate gyrus. In these microenvironments, astrocyte-like neural stem cells (NSCs) proliferate and produce neural precursors generating neuronal and glial progeny (Obernier and Alvarez-Buylla, 2019). Neurogenic niches host a heterogeneous cellular population that includes NSCs at different stages of development: quiescent NSCs (qNSCs), active NSCs (aNSCs), transit-amplifying progenitors (TAPs), and neuroblasts (NBs) (Bond et al., 2015; Obernier and Alvarez-Buylla, 2019). Single-cell transcriptome analyses of the mouse SVZ have revealed gene expression patterns characteristic for each of these stages but have also uncovered additional intermediate states (Beckervordersandforth et al., 2010; Codega et al., 2014; Llorens-Bobadilla et al., 2015; Dulken et al., 2017; Zywitza et al., 2018).

The knowledge on transcriptome-wide gene expression in the primate SVZa is limited. Most such studies have mapped gene expression in diverse brain regions during development and in the adult (Bernard et al., 2012; Hawrylycz et al., 2012; Miller et al., 2014; Bakken et al., 2015, 2016), but few studies have focused on gene expression in the neurogenic niches (Miller et al., 2013; Foret et al., 2014; Sandstrom et al., 2014). A comparison of the adult SGZ transcriptomes of mice and rhesus monkeys reported a conservation of the molecular signatures of precursors involved in hippocampal neurogenesis (Miller et al., 2014). Transcriptome analyses of isolated SVZa precursors from adult baboon monkeys revealed genes regulated by active (H3K4me3) or repressive (H3K9me3) epigenetic marks (Foret et al., 2014; Sandstrom et al., 2014).

Brain ischemia is a major cause of death and disability. Several experimental models have been used to study the response of the brain to ischemia, but only a few of those use non-human primates (Tajiri et al., 2013). In ischemic rodent models, the NSCs attempt to replenish lost or damaged cells by enhancing NSC proliferation followed by neuron production (Marques et al., 2019). The capacity of the primate brain to undergo reparative processes is more limited than in rodents (Alunni and Bally-Cuif, 2016). Understanding the molecular cause which underlies this difference between primates and rodents is an important step in the development of strategies for restoring human brain functions after injury (Jessberger and Gage, 2014). We have previously established a model of transient global ischemia in adult macaque monkey. Such ischemia induces regional tissue damage affecting neuronal populations in the hippocampus, striatum, neocortex and cerebellum (Yamashima et al., 1996; Yoshida et al., 2002). In this model, we have observed an enhanced proliferation of bromodeoxyuridine (BrdU) labeled progenitor cells in SVZa, in the second postischemic week (Tonchev et al., 2003, 2005).

To study gene expression changes preceding the onset of proliferation in the SVZa, we investigated the transcriptome-wide response of the SVZa niche following global brain ischemia. Using a combination of transcriptomics and high-throughput *in situ* hybridization (ISH), we examined the changes in gene expression in the SVZa and its subregions at postischemic day 7. We found that ischemic SVZa shows a transcriptional profile reminiscent of a profile typical for quiescent stem cells, astrocytes and oligodendrocytes. In addition, we determined by ISH the expression of 150 genes in control and ischemic SVZa, digital images are freely available online^1^. A comparative analysis of the expression pattern these 150 genes yielded several novel gene markers for SVZa cells, including the apelin receptor (*APLNR*).

## Materials and Methods

### Subjects

Experiments with the 7 monkeys used were approved by the Animal Care and Ethics Committee of Kanazawa University, Japan (Approval protocols AP-031498 and AP-080920). The monkeys (5 female and 2 male *Macaca fuscata*) were kept in air-conditioned cages and had free daily access to food and water. The monkeys were 4–6 years of age at the time of the experiments. Six monkeys were subjected to surgical procedures to induce global brain ischemia or to a sham operation, and sacrificed 7 days later. One additional monkey which was not operated received daily injections (100 mg/kg, *i.v.*) of 5-bromo-2’-deoxyuridine (BrdU, Sigma-Aldrich Corp., St. Louis, MO, United States) for 5 consecutive days. This monkey was sacrificed 2 h after the last BrdU injection.

Optimization of the ISH conditions were performed on sections from *Macaca mulatta* brain tissues obtained from the German Primate Center (Göttingen, Germany). All animals were offsprings of monkeys that have been bred in captivity. The animals were kept under the regulations for non-human primates by the guidelines for the accommodation and care of animals used for experimental and other scientific purposes (2007/526/EC; Appendix A ETS 123).

Mice were sacrificed according to the German Law on Animal Welfare. Sacrifice is licensed by the Veterinary authorities of Göttingen, Germany (392000_2a/Si/rö, 09/12/2013).

### Surgical Procedures and SVZa Microdissection

Three monkeys underwent transient whole brain ischemia, and 3 monkeys were subjected to a sham surgery (Yamashima et al., 1996; Yoshida et al., 2002; Tonchev et al., 2003, 2005). The brachiocephalic trunk and left subclavian arteries were clipped for 18 min (Supplementary Figure 1A). At day 7 after the sham or ischemic surgery, the monkeys were anesthetized with a lethal dose of sodium pentobarbital and intracardially perfused with 0.5L cooled sterile saline. Within less than 20 min from the start of the perfusion, craniotomy was performed and the whole brain was extracted, sectioned into two hemispheres through the corpus callosum, and each hemisphere was snap-frozen in liquid nitrogen and stored at −80°C. The right hemisphere was used for the subsequent analyses. In the monkey brain, the lateral ventricle spans more than 30 mm, between levels with atlas coordinates +35 mm rostrally to +2 mm caudally (Saleem and Logothetis, 2007). The right hemisphere was sectioned into 5 mm-thick coronal slices. The slice which included the anterior commissure and the head of the caudate nucleus (atlas coordinates +20 mm to +25 mm (Saleem and Logothetis, 2007) was placed under a stereomicroscope and three adjacent tissue fragments (∼1 mm × 0.2 mm, 3 mm thick) were dissected with a microscalpel along the striatal side of the right lateral ventricle (Supplementary Figures 1B1–B3). The callosal side of the lateral ventricle was not included in the excised tissue sample. All steps were performed on dry ice to avoid complete defreezing and RNA degradation within the samples. The dissected specimens were transferred to an Eppendorf tube, placed in liquid nitrogen and stored at −80°C until the RNA extraction step.

### RNA Sequencing (RNA Seq) and Data Analysis

#### RNA Seq

RNA Seq was performed at the Transcriptome and Genome Analysis Laboratory (TAL, University of Göttingen). The total RNA from each SVZa sample was extracted using the phenol-chloroform extraction protocol and 1 μg of total RNA was converted into Illumina sequencing libraries using the TruSeq RNA Sample Preparation Kit (Illumina, RS-122-2002). The size range of the final cDNA libraries were determined using Bioanalyzer 2100 (Agilent Technologies; Santa Clara, California, United States). The generated cDNA libraries were then amplified and sequenced using the cBot and HiSeq2000 (Illumina Inc., San Diego, California, United States) (SR; 1 × 50 bp; ∼ 30 million reads per sample). Finally, the sequence images were transformed to *per* read base call files (fastq files) using the Illumina software BaseCaller and CASAVA v1.8.2. RNA-Sequencing data have been deposited to GEO under the accession ID GSE136036.

We checked the RNA Seq data *via* FastQC quality control tool (v.0.10.0, Babraham Bioinformatics). Raw sequence reads were aligned to the *Macaca mulatta* genome (ensembl 73) by TopHat bioinformatics tool (V2.0.8 custom parameters: –no-coverage-search) (Trapnell et al., 2009). The gene annotation was downloaded from RhesusBase v2.0^2^. This database uses all published Macaque RNA-seq data to refine ensembl Macaque gene annotation. The reads number mapped to each gene were calculated using bedtools (v2.16.2 default parameters) (Quinlan and Hall, 2010).

In order to reduce the false discovery rate due to variations between monkeys, we applied two methods to identify genes differentially expressed after ischemia. First, read numbers were normalized to RPKM (reads per kilobase per million). Since the reads mapped to known transcripts can differ significantly, we used the total number of reads mapped to a known transcript to do normalization. We compared the 9 sham-operated and the 9 ischemic samples with the paired *T*-test to calculate the differentially expressed genes. Second, the R package DESeq was applied to normalize the reads counts and calculate the significance of the differential expression, taking the batch effect into account. The paired *T*-test *p*-value < 0.05, DESeq *p*-value < 0.05, and log2-transformed fold change > 0.5 were used as criteria to select the differentially expressed genes with an FDR = 2.1%. It was calculated as the ratio between the number of differentially expressed genes by chance, which is estimated by the median number of significant genes for 1000 random permutation of samples, and the number of significant genes of real data under the same criterion.

#### Functional Enrichment Analysis of Monkey SVZa-DE Genes

The lists of monkey SVZa-DE-UP or SVZa-DE-DOWN genes were uploaded to the DAVID functional annotation tool^3^. Then, representative enriched biological functional terms were selected. To perform the Gene Set Enrichment Analysis (GSEA) analysis, the combined list of SVZa-DE-UP and SVZa-DE-DOWN genes was uploaded to GSEA. Using FDR *q*-value < 0.05 as a cut-off generated 166 enriched biological and cellular component terms. The Fisher’s exact test was applied to identify the terms showing a statistically significant difference for the SVZa-DE-UP or the SVZa-DE-DOWN genes.

#### Protein-Protein Interaction Network

The combined list of monkey SVZa-DE-UP and SVZa-DE-DOWN genes was uploaded to the STRING database^4^, then protein-protein interaction from STRING were visualized by Cytoscape^5^.

### Non-radioactive Automated ISH

Automated ISH was performed on frozen sections (Lein et al., 2007). Coronal tissue blocks approx. 2 × 2 cm in size, which included the caudate nucleus (Supplementary Figures 1B1,B2), were dissected from the right hemisphere. The blocks were embedded in Tissue-Tek O.C.T (Sakura), quick frozen, and sectioned at 20-μm thickness. Hybridization was performed on a Tecan (Männedorf, Switzerland) ISH robot as previously described (Lein et al., 2007). Macaque monkey specific templates, 700–1000 nucleotides long, were synthesized. Primer and template and sequences are available online at http://www.monkey-niche.org. Digoxigenin-tagged riboprobes, were generated by *in vitro* transcription. Hybridized probes were detected by a two-step chromogenic catalyzed reporter deposition. For FISH staining, the hybridized probe for *APLNR* was visualized using fluorochrome-conjugated reagents. The mouse *Aplnr* mRNA (Entrez Gene reference: NM_011784.3) was visualized using SP6/T7 primers with a total length of the template 649 of nucleotides. The template was generated using the following specific primers: Forward: TCTAGGCACCACAGGCAATG; Reverse: GGTCACTACAAGCACCACGA.

### Visualization of Colorimetric ISH and Atlas Generation

After colorimetric ISH, slides were cover-slipped and digitally scanned at 0.501 μm/pixel using an Aperio ScanScope AT2 whole slide scanner (Leica Biosystems, Wetzlar, Germany). Images were cropped and stored in Aperio SVS format. Next the images were converted into a zoomable image pyramid format (Zoomify http://www.zoomify.com/) with Libvips (Martinez and Cupitt, 2005); https://libvips.github.io/libvips/, and the OpenSlide library (Satyanarayanan et al., 2013)^6^. The metadata was extracted from the glass slide label via Optical Character Recognition (Tesseract https://github.com/tesseract-ocr/tesseract), checked and stored in a MySQL Database together with the standard image data. The database is accessed using a web interface and the images are rendered via a custom implementation of the Openseadragon viewer^7^ which additionally features an image comparison mode. The atlas coordinates of the images shown in the database are according to Saleem and Logothetis, 2007. The atlas coordinates of the sections shown in Figures 3D–F, 4D–K, 5, 6C–F are provided in Supplementary Table S1.

### Quantitative Measurements of Colorimetric ISH

To quantify the gene expression in the SVZa, we used the custom software Celldetekt (version 2.7; Carson et al., 2005; https://github.com/tumrod/cellDetekt). Celldetekt is a python script^8^ that uses the Python Imaging Library^9^. The input images for analysis were derived from the *monkey-niche.org* database and imported into Celldetekt. The expression signal strength for ischemic sections was compared to the control and the fold increase is shown in the bar diagrams of Figures 3D–F, 4D–K, 5, 6C–F. Celldetekt locates the cells on the images and estimates the level of expression in each cell. The cells are classified according to the ISH precipitates in each cell as: (*i*) level 3 cells are filled with dye precipitate, (*ii*) level 2 cells are partially filled with precipitate, (*iii*) level 1 cells are characterized by scattered minute particles of deposit, and (*iv*) level 0 cells lack detectable precipitate (Carson et al., 2005). The cells in the RoIs (CSVZ and SEL) were evaluated within an area of 100 μm in width x 1500 μm in length, starting from the dorsal tip of the lateral ventricle (Supplementary Figure 2). The cells in the EL were evaluated within the thickness of the ependymal layer along a length of 1500 μm, starting from the dorsal tip of the lateral ventricle. The number of level 2 and 3 cells out of the number of all cells was calculated as a ratio for one sham-operated and one postischemic SVZa.

### Immunohistochemistry

Immunofluorescence stainings of monkey specimens were performed following the FISH labeling of *APLNR*. The slides were washed 3 × 5 min in PBS and subjected to antigen retrieval using the Vector Antigen Unmasking Solution (Cat. No. H-3300, Vectorlabs, Burlingame, CA, United States). The slides were incubated in a microwave oven at 800 W for 3 × 5 min, each cycle being followed by a cooling step on ice for 30 min. The slides were then washed in PBS 3 × 5 min and blocked for 1 h in 10% normal goat serum (Cat. No S-1000, Vectorlabs) in PBS with 0.1% Triton-X100. The primary antibodies were applied in blocking solution overnight at 4°C. On the next day, the sections were washed in PBS 3 × 5 min and incubated for 2 h at room temperature in the respective secondary antibody conjugated to AlexaFluor-488 or AlexaFluor-647 fluorochromes (Thermo Fisher Scientific, Germany). The following primary antibodies were used: rat anti-BrdU (1:100, Cat. No Ab6326, Abcam, Cambridge, United Kingdom); mouse anti-GFAP (1:400; Cat. No M0761, Dako-Agilent Technologies GmbH, Hamburg, Germany); chick anti-GFAP (1:1000; Cat. No AB5541, Merck Millipore); rabbit anti-GLUT1/SLC2A1 (1:100; Cat. No HPA031345, Sigma-Aldrich); mouse anti-VIM (1:1000; Cat. No MAB3400, Merck Millipore).

### Microscopy on Fluorescently Labeled Sections and Image Analysis

The slides labeled by FISH and immunofluorescence were imaged using a fully motorized wide-field epifluorescence microscope Zeiss AxioImager Z.2 (Carl Zeiss GmbH) with an AxioCam Mrm rev.3 monochrome CCD camera (Carl Zeiss GmbH), and AxioVision v.4.9 software. A set of 3–5 non-overlapping randomly selected Z-stacks within the SEL of each section were captured through an EC Plan-Neofluar objective 40×/0.75 at resolution of 0.325 μm/pixel and Z-axial resolution of 0.55 μm. The number of sections visualized and the number of monkeys from which data are derived are listed in Supplementary Table S14.

The camera was set to a binning factor 2 × 2 to reduce both scanning time and camera noise. Shading from the irregular illumination field was corrected during the acquisition via the camera built-in shading correction using prerecorded illumination field images from fluorescent test slides. All camera settings for the different channels were kept constant. The exposure time was allowed to vary only when this was needed to avoid saturation. The images were saved.zvi format (Zeiss) of the AxioVision software, stitched together, and exported to a 16 bit TIFF format for further processing and analysis as described in the following section.

### Quantitation of Cells on Sections Labeled by Multiple Fluorescence Dyes

Because of the large size of the primate SEL, analysis of cell identity required screening thousands of cells. We therefore developed a semi-automated digital image cytometry workflow (Wählby, 2003; Chieco et al., 2013). The process includes the following steps: (1) Preprocessing, including channel extraction and image denoising, (2) Top-Hat morphological filtering for improving signal/noise ratio, (3) Nuclear segmentation, (4) Channel thresholding, (5) Classification by a pretrained decision tree and counting of the RoIs. This algorithm for the analysis of co-expression might be useful to those who aim at scanning entire brain regions in large-sized brains, such as the primate brain.

The goal of the workflow was to reduce the operator introduced bias by creating an automated algorithm for image processing and analysis. We applied a unified, reproducible approach to a set of images with variable brightness distributions of the signal or the background. The workflow includes the following major steps following the image acquisition:

#### Preprocessing

Generate a projection of the z-stack on one all-in-focus plane using the Smooth manifold extraction (SME) algorithm (Shihavuddin et al., 2017) as implemented in the SME-projection plugin for FIJI/ImageJ^10^. Perform image denoising using the Non-Local Means algorithm as implemented in the Fiji/ImageJ software for image processing and analysis (Buades et al., 2011; Schindelin et al., 2012); channel extraction and sorting into one of the following channel classes: nuclear antigen, cytoplasmic antigen, mRNA FISH cytoplasmic channel, or DAPI channel.

#### Top-Hat Morphological Filtering

Top-Hat morphological filtering by reconstruction of each channel to suppress background and improve the signal-to-noise (S/N) ratio. The Top-Hat (“top minus hat”) procedure subtracts an estimate of the background developed by a morphological opening from the original image. To eliminate the influence of dark background variability we added a step of morphological closing with a structuring element of the same size before the opening. This eliminates dark variations and the final image includes only the bright parts in the image that stand above the background. Instead of the classical gray-scale morphological filtering we used reconstructions of the opening and the closing thus reducing further image artefacts arising from the anisotropy of the structuring elements (SEs) (Soille, 1999; Legland et al., 2016). Each channel class (nuclear antigen, cytoplasmic antigen, mRNA FISH cytoplasmic channel, or DAPI channel) was filtered with a disk-shaped SE, of size appropriate for the distributions of signals. The sizes were selected with the help of digital granulometry of the preprocessed channel images (Legland et al., 2016).

#### Definition of Cellular Masks

After the top-hat filtering the DAPI channels were processed with the CellProfiler 3 (Bray et al., 2015) to detect, declump and export binary masks of nuclear connected regions. The unprocessed images were used to manually select areas 150 μm below the ependymal layer. All nuclear masks within these bounds were extracted, dilated under restriction to avoid overlap by ∼3 μm and saved as collections of labeled cellular masks for the analysis of the cells in each image.

#### Channel Thresholding

The images for analysis were thresholded in two steps. First the top-hat images were Z-transformed – the gray value of each pixel was replaced by its Z-score and all pixels with values ≤ 1, i.e., less than the mean plus one standard deviation, were set to 0. Next these images were thresholded with Li’s Minimum Crossenthropy algorithm (Li and Tam, 1998) as implemented in Fiji/ImageJ.

#### Automatic Classification and Counting of the Cellular Labels

We used a simple decision tree which classifies the cellular labels into positive and negative considering the fraction of the area covered by the corresponding thresholded signal. The limits for acceptance of positivity were selected after a receiver operating characteristic (ROC) analysis of ∼300 cells classified by an experienced researcher. All used imageJ macro scripts and the steps of CellProfiler pipeline are available in an online github repository^11^.

## Results

### The Transcriptome Changes in the SVZa Niche Following Ischemia

**FIGURE 1:**
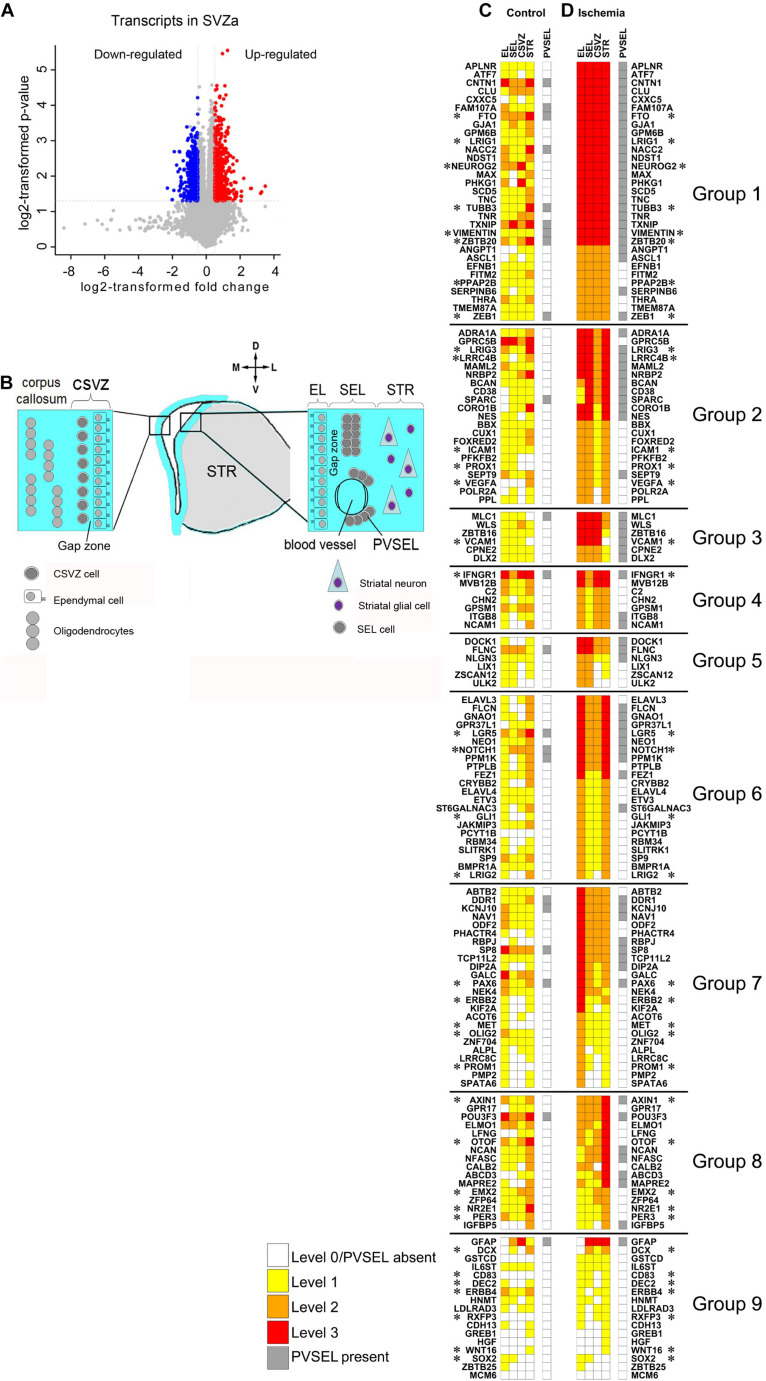
Molecular changes in the neurogenic niche induced by ischemia. **(A)** Volcano plot displays changes in gene expression in monkey SVZa. Transcripts up-or down-regulated and those unchanged are in red or blue and gray. **(B)** Cellular architecture of SVZa on the striatal and the callosal side of the lateral ventricle. For details see Text. **(C,D)** Heat maps illustrating gene expression changes of 150 genes in EL, SEL, CSVZ, STR and PVSEL upon ischemia. The 36 genes implicated in stem cell biology are marked (*). Original data are on “monkey-niche.org.” Expression of 150 genes, deposited in the “monkey-niche” database (Supplementary Table S7) is shown for five regions of interest (Figure 1B). The genes in **(C,D)** are grouped according to their postischemic upregulation: Group 1 (markedly enhanced expression levels in all RoIs), Group 2 (predominantly upregulated in the RoIs on the striatal side of the ventricle), Group 3 (strongly induced in EL, SEL and CVSZ), Group 4 (strongly induced in EL, CSVZ and STR), Group 5 (strongly induced in EL and SEL), Group 6 (strongly induced in EL and STR), Group 7 (strongly induced in EL), Group 8 (strongly induced in STR), Group 9 (genes with low level of expression or not belonging to the other groups).

**FIGURE 2:**
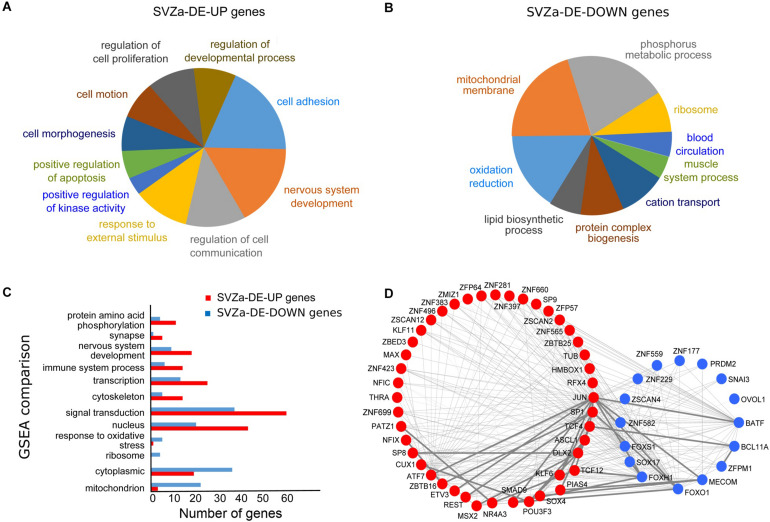
Bioinformatics analysis of the genes differentially induced after ischemia in monkey SVZa. **(A,B)** Pie charts showing the Gene Ontology (GO) categories for SVZa up-regulated genes (SVZa-DE-UP) and SVZa down-regulated genes (SVZa-DE-DOWN). Only those GO categories are shown for which there is enrichment relative to the total SVZa genes (all genes expressed in the SVZa). The SVZa-DE-UP and SVZa-DE-DOWN genes for each GO category are listed in Supplementary Table S4. **(C)** GSEA comparison for SVZa-DE-UP *versus* SVZa-DE-DOWN genes. Fisher’s exact test was applied to identify the sets that show difference between SVZa-DE-UP *and* SVZa-DE-DOWN genes. The genes that correspond to each category are listed in Supplementary Table S5. **(D)** Protein interaction network of the differentially expressed transcription factors. The red nodes represent SVZa-DE-UP genes, while the blue nodes represent SVZa-DE-DOWN genes. The thin lines indicate low interaction score (<0.4), while the thick lines indicate medium or high interaction score (≥0.4).

To uncover the changes in gene expression upon ischemia in the macaque SVZa niche, we performed transient global cerebral ischemia in adult macaque monkeys. In both sham-operated and ischemic tissue ∼25,000 transcripts corresponding to ∼16,000 genes were detected (Supplementary Table S2). Upon ischemia a total of 1554 transcripts were differentially expressed (DE), 816 (541 genes) of which were upregulated (SVZa-DE-UP genes) and 738 transcripts (488 genes) were down regulated (SVZa-DE-DOWN genes) (Figure 1A and Supplementary Table S3). Gene ontology (GO) and gene set enrichment analysis (GSEA) revealed that SVZa-DE-UP and SVZa-DE-DOWN genes encompass distinct functional groups (Figures 2A–C). One third (185) of the SVZa-DE-UP genes were associated with GO biological process categories that were over-represented when compared to the total SVZa genes. These SVZa-DE-UP genes were enriched in “cell adhesion”, “nervous system development”, “regulation of cell communication”, “response to external stimulus”, and “cell proliferation” (Figure 2A and Supplementary Table S4). A GSEA analysis showed enrichment in SVZa-DE-UP genes, in the categories “signal transduction”, “nervous system development”, “cytoskeleton”, and “protein amino acid phosphorylation” (Figure 2C and Supplementary Table S5).

We looked for transcriptional regulators, which were differentially expressed in SVZa after ischemia. We identified 45 upregulated transcription factors corresponding to 18 protein families and 17 down-regulated transcription factors corresponding to 8 protein families (Supplementary Table S6). C2H2 type zinc finger protein family was the most enriched protein family, with 18 upregulated and 11 down-regulated genes, followed by bHLH and homeobox protein families with 5 upregulated genes each. Next we determined which of the transcription factors interact physically or functionally using the STRING database (see foot note 4). This revealed a highly interconnected network formed by both upregulated and down-regulated factors (Figure 2D). Several transcription factors formed a network hub, including *JUN*, *DLX2*, *ASCL1*, *SOX4*, *BATF*, *FOXS1*, *SOX17*, and *FOXH1* with *JUN* in the center of the network. This raises the possibility that the components of this transcription factor network are involved in regulating aspects of the response of the SVZa to ischemia. Such *in silico* interaction data need to be tested for physical or functional interaction.

In the group of the 488 SVZa-DE-DOWN genes, GO analysis revealed that 143 genes were over-represented in the categories “oxidation reduction,” “mitochondrial membrane,” “cation transport,” and “ribosome” (Figure 2B and Supplementary Table S4). Consistent with the GO results, the GSEA analysis showed enrichment of the gene groups “response to oxidative stress”, and “mitochondrion” (Figure 2C and Supplementary Table S5). The genes annotated by these GO terms typically included genes involved in mitochondrial function and their down-regulation following ischemia most probably reflects the dysfunction of mitochondria under hypoxic conditions (Solaini et al., 2010).

### *In situ* Analysis of Gene Expression Changes After Ischemia

The transcriptomic analysis provides a global view of which gene expression levels change after an ischemic insult. To anchor this change to cells and tissues in their natural environment we performed colorimetric ISH on coronal sections through the SVZa (Supplementary Figures 1B1,B2). We generated 150 macaque-specific riboprobes that were hybridized to sections using a flow-through robotic system (Lein et al., 2007) to minimize experimental variability. ISH data from control and ischemic brain sections are freely available on a public database (www.monkey-niche.org; Supplementary Figure 1C). Of note, the images on www.monkey-niche.org show gene expression the SVZa but also in additional brain regions, including the corpus callosum, the cingulate gyrus and the thalamus. For identification of such structures see Saleem and Logothetis, 2007.

In the monkey, the SVZa is composed of the ependymal layer (EL) and the subependymal layer (SEL). The SEL is separated from the EL by an approximately 100-μm-wide, cell-sparse gap zone (Figure 1B; Gil-Perotin et al., 2009). Underneath the striatal SEL lies the striatal parenchyma (STR). NSCs reside predominantly in the SEL on the striatal side of the lateral ventricle (Fuentealba et al., 2015). The callosal side of the monkey lateral ventricles contains a few proliferating cells under the ependyma which do not form clusters (Tonchev et al., 2005). We refer to this region as callosal SVZ (CSVZ**;** Figure 1B). In addition, there are perivascular cell clusters in the striatal SEL (PVSEL). The level of expression in the regions of interest (RoIs) EL, SEL, CSVZ, and STR was visually graded as level 0 (not detected), level 1 (low), level 2 (moderate) or level 3 (high) expression. Expression in PVSEL was assessed as “present” or absent (for illustrations of grading see Supplementary Figure 3).

In this report we focus on genes that belong to the SVZa-DE-UP group because this group was enriched for genes that relate to progenitor cell biology (Figure 2A). From the 541 SVZa-DE-UP genes, we performed ISH for 114 genes from the top 300 highly expressed genes in the transcriptome, and which relate to stem cell biology (signaling pathways, transcription factors, regulators of cell adhesion, etc.). Additionally, we included 36 genes for the ISH analysis because they are implicated in stem cell biology (Supplementary Table S7). Of the 114/36 SVZa-DE-UP genes the visual scoring revealed enhanced postischemic expression in at least one RoI for 102/27 genes. A heat map compiled from the data for the annotated 150 genes visualizes the marked effect of ischemia on the gene expression patterns (Figures 1C,D). Genes whose expression is strongly enhanced in all RoIs are found on top of the heat map (Figure 1D), genes induced strongly in two RoIs are found in the middle of the map. Weakly induced genes are found toward the bottom of the map. The genes in the top half of the “ischemia” (Figure 1D) map show strongly enhanced expression in all RoIs, including the STR, suggesting that ischemia considerably affects striatal cells as well. Most of the genes are induced in the SEL, the main stem cell-containing region. Whenever a gene is strongly expressed in the SEL, it is also strongly expressed in 2 or 3 additional RoIs (EL, CSVZ or STR).

### Enrichment of Astrocytic and Oligodendrocytic Transcription Profiles in SVZa-DE-UP Genes

**FIGURE 3:**
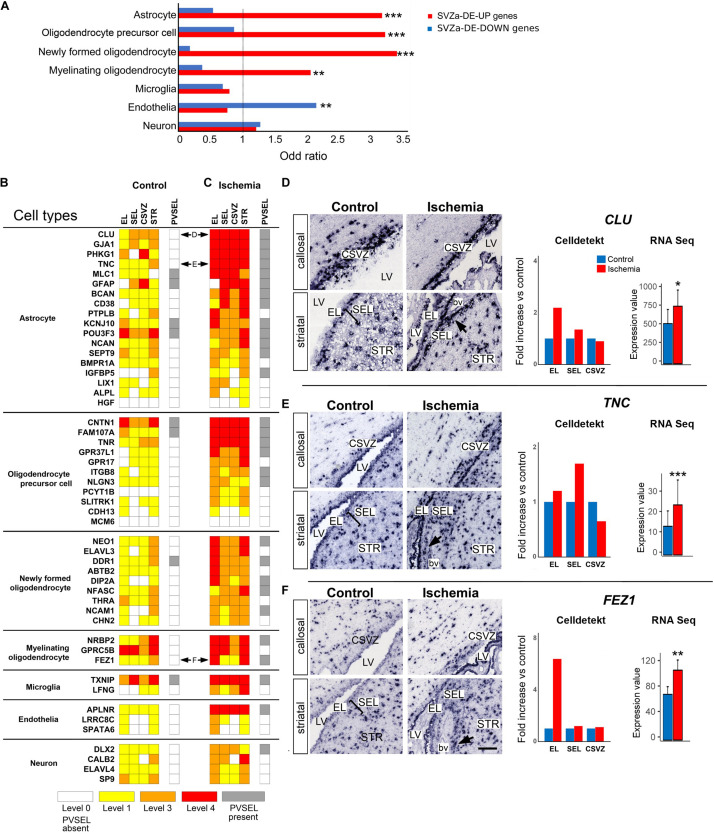
Upregulation of cell-type specific genes in SVZa after ischemia. **(A)** Relative to the total SVZa genes, the SVZa-DE-UP were enriched for the transcription profiles of astrocytic or oligodendrocytic markers (Zhang et al., 2014). Monkey SVZa-DE-DOWN genes were significantly enriched for endothelial genes. * Fisher’s exact test *p*-value < 0.05; ** Fisher’s exact test *p*-value < 0.01; *** Fisher’s exact test *p*-value < 0.001. **(B,C)** Comparative heat maps showing differential expression of genes – assessed by visual scoring -which are characteristic for the different cortical cell types listed on the left. Horizontal double arrows refer to ISH data shown in **(D–F)**. **(D–F)** ISH data illustrating expression of selected genes that are characteristic for astrocytes (**D**, *CLU*; **E**, *TNC*) or myelinating oligodendrocytes (**F**, *FEZ1*). Black arrows point at PVSEL. Expression of each gene was quantified in EL, SEL, and CSVZ (right column) using the Celldetekt. A bar plot shows the expression value of the respective gene as determined by RNA Seq (number of copies normalized by total count derived from 3 control and 3 ischemic brains); *p*-values are as in Panel **A**. See Supplementary Table S1 for atlas coordinates of the sections shown in panels **(D–F)**. Scale bar 100 μm.

The adult SVZ niche consists of several cell types (Martínez-Cerdeño and Noctor, 2018). In order to gain insight into the cell identity in our SVZa samples, we compared the transcriptome profiles of monkey total SVZa genes or SVZa-DE genes with the transcription profiles of defined cell types isolated from the adult mouse cortex (Zhang et al., 2014). We found that SVZa-DE-UP genes were significantly enriched for genes typical for oligodendrocytic cells or astrocytes (Figure 3A and Supplementary Table S8). In contrast, SVZa-DE-DOWN genes were enriched for genes typical for endothelial cells (Figure 3A).

**FIGURE 4:**
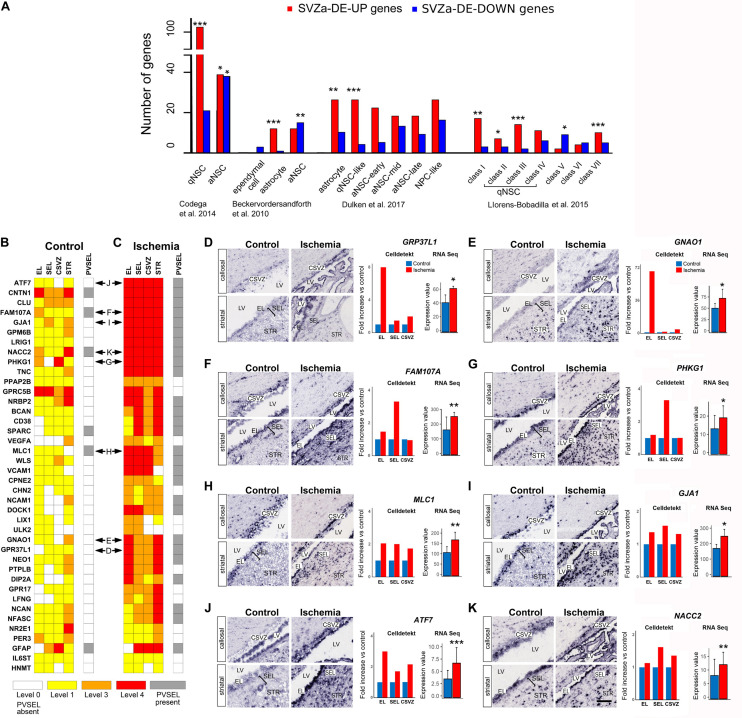
Prevalence of transcripts characteristic for quiescent NSCs in ischemic SVZa. **(A)** Monkey total SVZa genes or SVZa-DE genes were compared with the gene datasets enriched in mouse SVZ progenitors (Beckervordersandforth et al., 2010; Codega et al., 2014; Llorens-Bobadilla et al., 2015; Dulken et al., 2017). The enrichment for each of the progenitor transcription profiles in monkey SVZa-DE genes was statistically analyzed. Monkey genes that correspond to each progenitor type are listed in Supplementary Tables S9–S12. * Fisher’s exact test *p*-value < 0.05; ** Fisher’s exact test *p*-value < 0.01; *** Fisher’s exact test *p*-value < 0.001. **(B,C)** Comparative heat maps of 40 genes from the “monkey-niche.org” database which are found in the datasets of Codega et al., 2014; Dulken et al., 2017; Llorens-Bobadilla et al., 2015. Horizontal double arrows refer to ISH data shown in **(D–K)**. **(D–K)** ISH data, Celldetekt-based quantification of ISH and RPKM based quantification revealed a region-specific strong post-ischemic enhancement in the EL (**D**, *GPR37L1*; **E**, *GNAO1*) or in the SEL (**F**, *FAM107A*; **G**, *PHKG1*). Other genes **(H–K)** were enhanced in EL, SEL and CSVZ (*MLC1*, *GJA1*, *ATF7*, and *NACC2*). Gene expression was quantified (right column) using Celldetekt and transcriptomic data as described in Figure 3. See Supplementary Table S1 for atlas coordinates of the sections shown in panels **(D–K)**. Scale bar 100 μm.

For 50 of the cell type-enriched genes, ISH data are available in the “monkey-niche” database, and their expression strength was visually scored. A comparison of the heat maps of control (Figure 3B) and ischemic (Figure 3C) SVZa shows that 34 out of these 50 genes were upregulated after ischemia in three of four RoIs. In addition, 29/50 genes showed an increased expression in perivascular cell clusters (Figure 3C, gray boxes; arrows in Figures 3D–F). Among the astrocytic genes upregulated by ischemia were *CLU* (Figure 3D), *TNC* (Figure 3E), *PHKG1* (Figure 4G) and *GJA1* (Figure 4I). Among the enhanced oligodendroglial genes were *FEZ1* (Figure 3F), *GPR37L1* (Figure 4D) and *FAM107A* (Figure 4F). Quantification of expression using Celldetekt software was consistent with the visual scoring shown in Figures 3B,C. A bar plot shows the expression value of the respective genes that are extracted from the RNA Seq data. In most cases, there is a good agreement between RPKM data, Celldetekt-analysis and visual assessment. Altogether, the transcriptomic and ISH analyses demonstrate an upregulation of astrocytic or oligodendrocytic transcripts in postischemic monkey SVZa.

### Predominance of qNSC-Related Gene Signatures in the Ischemic Primate SVZa

The progenitor pool in adult mouse SVZ represents a heterogeneous population with subtypes, including the quiescent NSCs (qNSCs) which are slowly proliferating, active NSCs (aNSCs) that further proliferate and produce multipotent transit-amplifying progenitors (TAPs). The transcription profiles of the progenitors at each of these stages have been characterized, in the mouse SVZ, using single-cell RNA Seq (Beckervordersandforth et al., 2010; Codega et al., 2014; Llorens-Bobadilla et al., 2015; Dulken et al., 2017).

To search for the presence of gene signatures typical for progenitor subtypes in the monkey SVZa-DE data sets, we compared our data (total SVZa genes or SVZa-DE genes) with the transcription profiles of mouse SVZ subtypes. A comparison with the profiles of murine qNSCs and aNSCs (Codega et al., 2014) revealed a significant over-representation of transcripts (102 genes) typical for qNSCs (Figure 4A and Supplementary Table S9). Only 39 SVZa-DE-UP genes belonged to murine aNSCs signature. Unlike the SVZa-DE-UP genes, the SVZa-DE-DOWN genes were enriched for aNSC signatures (Figure 4A and Supplementary Table S9). An enrichment of qNSC-like transcriptional profiles in monkey SVZa-DE-UP genes and of aNSC-like profiles in monkey SVZa-DE-DOWN genes was also observed when we compared the SVZa-DE transcripts with the datasets of two other studies (Beckervordersandforth et al., 2010; Dulken et al., 2017; Figure 4A and Supplementary Tables S10, S11).

Llorens-Bobadilla et al. (2015) have reported transcriptomes of murine SVZ progenitors isolated from control mice and mice subjected to global brain ischemia after 2 days. The authors identified gene signatures for seven subclasses of SVZ cells. Class I genes are characteristic for oligodendrocytes, classes II-VI characterize sequential NSC transitory states (dormant qNSC [class II)], primed qNSC [class III)], non-mitotic aNSC [class IV], mitotic aNSC [class V], TAPs [class VI], and class VII characterize neuroblasts. The induction of global cerebral ischemia to the mouse brain upregulated classes I, II and III (Llorens-Bobadilla et al., 2015). A comparison of our data sets with their study revealed that 38 of the monkey SVZa-DE-UP genes were enriched in genes of classes of I, II and III (Figure 4A and Supplementary Table S12) corresponding to gene signatures of oligodendroglial cells and qNSCs. We also observed that 10 of the up-regulated monkey genes showed enrichment in class VII (neuroblast) genes of Llorens-Bobadilla et al., 2015. These results raise the possibility that besides the predominance of the quiescent state of the SVZa cells, a small number of SVZa precursors leave quiescence.

40 of the 116 genes present in the qNSC datasets of mouse SVZ (Codega et al., 2014; Llorens-Bobadilla et al., 2015; Dulken et al., 2017) are available in the “monkey-niche” database. Notably, 23 of these qNSC-typical genes showed ischemia-induced upregulation in three or four of the RoIs (Figures 4B,C). In addition, 28 genes showed an increased expression in perivascular cell clusters along SVZa vessels (Figure 4C, gray boxes). In Figures 4D–K we show sample ISH data for genes with a specific pattern of postischemic upregulation. *GPR37L1* and *GNAO1* are predominantly upregulated in the EL after ischemia (Figures 4D,E). The genes *FAM107A* and *PHKG1* are strongly upregulated in the SEL and to a lesser extent in the EL (Figures 4F,G). The genes *MLC1, GJA1*, *ATF7*, and *NACC2* (Figures 4H–K) are upregulated in all three RoIs (EL, SEL, and CSVZ). The transcriptomics and ISH results support the notion of an enhancement of qNSC-related transcripts in monkey EL and SEL.

### Enhanced Postischemic Expression of Genes Belonging to Signaling Pathways

**FIGURE 5:**
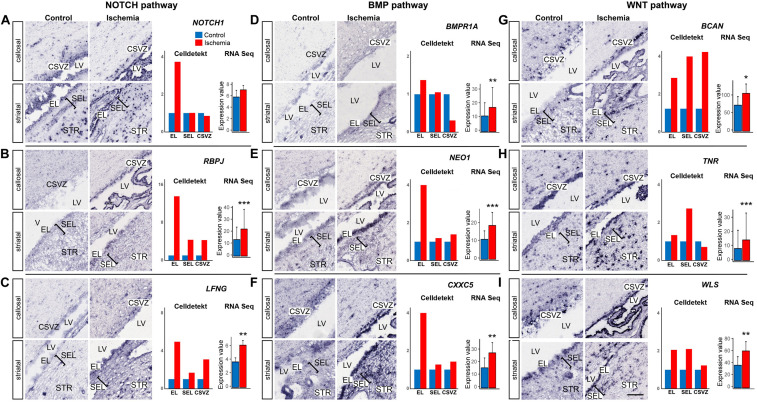
Post-ischemic increase of expression of genes of NOTCH, BMP and WNT pathways. **(A–C)** NOTCH pathway: *NOTCH1*, *RBPJ* and *LFNG*. Note the strong post-ischemic enhancement of these three genes in the EL. **(D–F)** BMP pathway: *BMPR1*, *NEO1* and *CXXC5*. Note the post-ischemic enhancement of the genes in the EL. **(G–I)** WNT pathway: *BCAN*, *TNR* and *WLS*. Note the post-ischemic enhancement of the genes in EL, SEL and for BCAN also in the CSVZ. Gene expression was quantified (right column) using Celldetekt and transcriptomic data as described in Figure 3. See Supplementary Table S1 for atlas coordinates of the sections shown in panels **(A–I)**. Scale bar 100 μm.

GSEA enrichment analysis revealed that components of several signaling pathways (NOTCH, BMP, and WNT) were significantly enriched in the SVZa-DE-UP genes (Supplementary Table S13). *NOTCH1* (Figure 5A), its transcriptional effector *RBPJ* (Figure 5B) and *LFNG* (Figure 5C), a regulator of Notch signaling in NSCs (Semerci et al., 2017) are strongly induced in the EL. BMP pathway-related transcripts, *BMPR1A* and *NEO1* receptors (Figures 5D,E) as well as the *CXXC5* zinc finger protein (Figure 5F) are also upregulated in the EL. Upregulation of WNT pathway members was evident in EL, SEL and CSVZ (Figures 5G–I). In contrast to the SVZa-DE-UP genes, the SVZa-DE-DOWN genes were not significantly enriched for genes belonging to these pathways (Supplementary Table S13).

### Novel Gene Markers for the Primate SVZa

**FIGURE 6:**
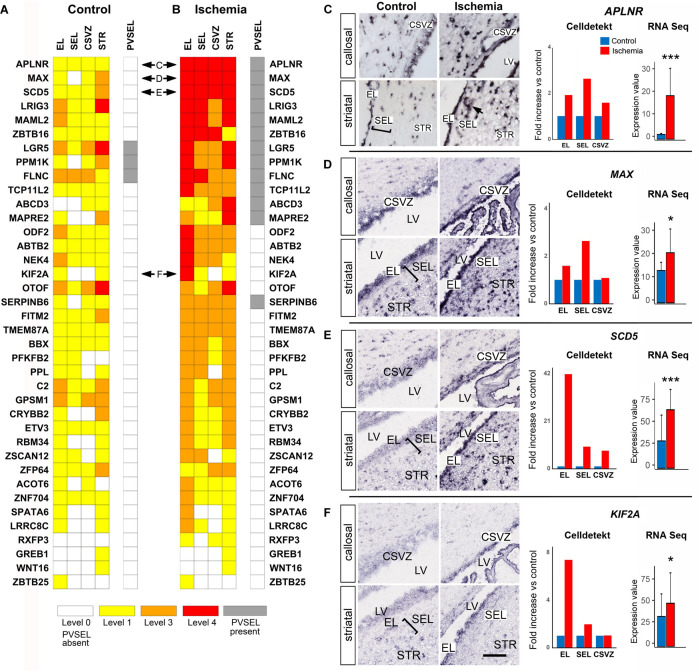
Expression patterns of genes not previously documented in SVZ niche. **(A,B)** Heatmaps of control and ischemic SVZa. 38 genes are arranged according to the strength of postischemic expression. Horizontal double arrows refer to ISH data shown in **(C–F)**. **(C–F)** Strong enhancement of *APLNR* in both EL and SEL **(C)**. Note *APLNR* expression in SEL cells adjacent to blood vessels (arrow). The gene *MAX* **(D)** is enhanced in both EL and SEL, while *SCD5* **(E)** and *KIF2A* **(F)** are predominantly up-regulated in the EL. Gene expression was quantified (right column) using Celldetekt and transcriptomic data as described in Figure 3. See Supplementary Table S1 for atlas coordinates of the sections shown in panels **(C–F)**. Scale bar 100 μm.

Single-cell RNA Seq experiments have provided transcriptional profiles of many SVZ cell types (Beckervordersandforth et al., 2010; Codega et al., 2014; Llorens-Bobadilla et al., 2015; Dulken et al., 2017; Zywitza et al., 2018). We found that 38 out of the 150 genes of the “monkey-niche” database were not present in any of these profiles (Figures 6A,B). Examples include *APLNR*, *MAX*, *SCD5*, and *KIF2A* (Figures 6C–F). The apelin receptor (APLNR; Figure 6C) is an angiogenesis-promoting cell surface molecule (Wu et al., 2017). Its expression was markedly increased upon ischemia in the EL as well as in non-vascular cells of the SEL (Figure 6C, arrow). Similarly, *MAX*, a transcription factor which is an obligate partner of the proliferation driver MYC, was also markedly enhanced after ischemia, particularly in the SEL (Figure 6D). *SCD5* (Figure 6E), encoding a primate-specific isoform of Stearoyl-CoA Desaturase (Wang et al., 2005), and the microtubule-associated protein *KIF2A* (Figure 6F) were both strongly upregulated in the EL after ischemia. Other interesting examples of novel SVZa genes are accessible on monkey-niche database and include the intestinal stem cell marker LGR5 (Barker et al., 2007) and the hematopoietic progenitor marker *ZBTB16* (Pardrige et al., 1990).

### Apelin Receptor Marks Specific Cell Populations in the Primate SVZa

**FIGURE 7:**
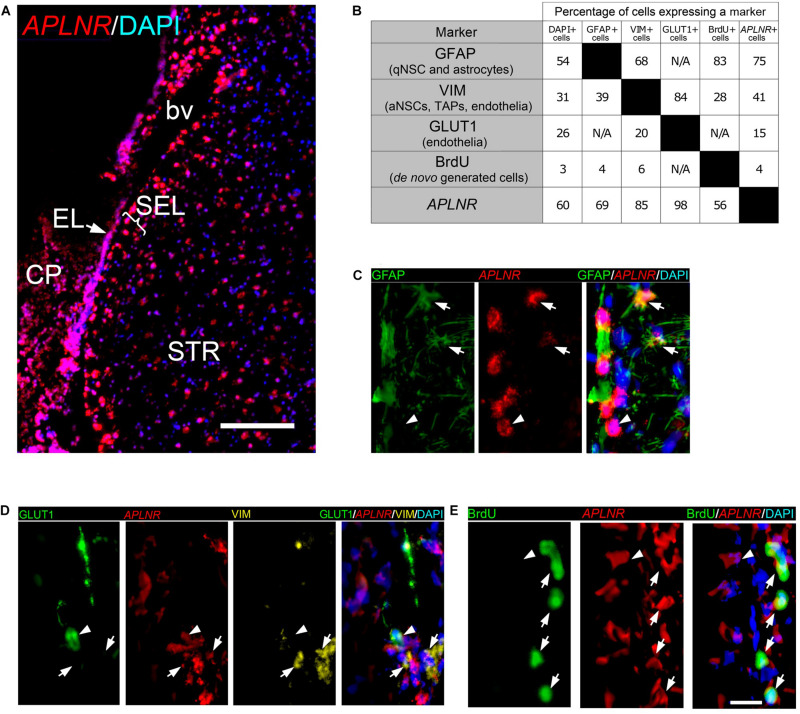
Cell identity of *APLNR* positive cells in SVZa of monkey brain. **(A)** Fluorescent ISH (FISH) staining of *APLNR* (red) on a coronal macaque brain section. A low magnification view of the striatal aspect of SVZa shows expression in EL, SEL and STR. **(B)** Two-way matrix showing the cell fractions expressing each of the markers analyzed. Each column corresponds to a SEL cell class positive for the corresponding marker. **(C–E)** High magnification views of SEL showing combinatorial labeling of *APLNR*, and GFAP, VIM, GLUT1 or BrdU. The EL is not shown. **(C)** *APLNR*/GFAP co-staining reveals the presence of *APLNR* + /GFAP + cells (arrows) and *APLNR* + /GFAP- cell (arrowhead). **(D)** *APLNR*/VIM/GLUT1 triple labeling in SEL. Arrows, *APLNR* + /VIM + /GLUT1- cells; arrowhead, *APLNR* + /VIM + /GLUT1 + cell. **(E)** *APLNR*/BrdU co-staining reveals the presence of *APLNR* + /BrdU + cells (arrows) and *APLNR* + /BrdU- cell (arrowhead). Scale bars 500 μm **(A)**, 25 μm **(E)**.

The transcriptome data showed ∼10-fold increase in the expression of *APLNR* (Figures 1C,D, 6C), one of the genes not previously documented in the rodent or primate SVZ. The ISH confirmed a strong induction of *APLNR* in postischemic monkey EL and SEL (Figure 6C). In the brain, Aplnr is expressed in vascular cells and neurons (Wu et al., 2017). Recent data indicate that Aplnr promotes postischemic angiogenesis and neuroprotection, at least in rodents (Wu et al., 2017). To identify which SEL cell type(s) express *APLNR* in monkey SVZa, we combined fluorescent ISH (FISH) for *APLNR* (Figure 7A) with immunofluorescence staining for markers of cells present in the niche (Figure 7B and Supplementary Table S14). One of the monkeys had been injected with the thymidine analog bromodeoxyuridine (BrdU) that selectively labels *de novo* generated cells (Tonchev et al., 2003, 2005).

To analyze the cell identity of thousands of cells in the primate SEL we developed a semi-automated approach. It involved segmentation of all DAPI stained nuclei, of cytoplasmic florescence signals derived from FISH or fluorescence-tagged antibodies against GFAP, VIM or GLUT1, and of nuclear BrdU. We found that 60% of all SEL cells express *APLNR* and 54% of all SEL cells express GFAP. A quarter of the SEL cells expressed VIM or GLUT1. After BrdU infusion, 3% of the SEL cells are BrdU-positive (BrdU+), confirming that only a few primate SEL cells were *de novo* generated (Figure 7B).

Examples of co-expression of *APLNR* with cell-type markers in the SEL (Figures 7C–E) and the percentages of co-expression (Figure 7B) are shown. 75% of the *APLNR*+ cells expressed GFAP (Figures 7B,C, arrows) and 69% of GFAP+ cells expressed *APLNR* (Figure 7B). These data show that *APLNR* is expressed in a significant proportion of SEL cells representing either niche astrocytes or qNSCs. *APLNR*/VIM co-labeling experiments demonstrated that 41% of the *APLNR*+ cells expressed VIM (Figures 7B,D, arrows), and 85% of the VIM+ cells were positive for *APLNR* (Figure 7B). To distinguish between *APLNR*+ /VIM+ endothelial cells and *APLNR*+/VIM+ neural progenitors (aNSCs or TAPs), we performed triple-labeling experiments combining *APLNR*/VIM co-staining with labeling for the endothelial marker GLUT1 (Pardrige et al., 1990; Figure 7D). We found that 98% of the GLUT1+ cells also expressed *APLNR* (Figures 7B,D, arrowhead). However, only 15% of the *APLNR*+ cells in the SEL were positive for GLUT1 (Figure 7B). Thus, we clearly detected *APLNR*+/VIM+ cells, which were negative for GLUT1 in SEL (Figure 7D, arrows), probably representing activated progenitors. To further support the notion that *APLNR* is expressed by proliferating cells, newly generated cells were labeled by BrdU infusion at five subsequent days. Double-staining for *APLNR* and BrdU revealed that 4% of the *APLNR*+ cells had incorporated BrdU (Figures 7B,E, arrows), and 56% of the BrdU+ cells expressed *APLNR* (Figure 7B). Notably, APLNR expression was evident in the SVZ niche not only upon ischemia, but also in the brain of sham-operated macaques. In contrast, in the mouse SVZ *Aplnr* mRNA was not expressed in the EL and only weakly expressed in the SEL (Supplementary Figure 4B, dotted lines). Strong *Aplnr* signal was detected in the murine neurons (Supplementary Figure 4B, arrowheads).

## Discussion

### Status of Neurogenic Niche One Week After Ischemia

To identify the response of the SVZ neurogenic niche upon ischemic insult in the adult primate brain is a challenging task. Here, we present the first comprehensive characterization of the molecular responses of the monkey SVZ at a transcriptome level seven days after an ischemic injury. The time point of our analysis precedes the proliferative response of the SVZ niche (Tonchev et al., 2005) by a week. We provide evidence that the predominant progenitor expression profile of SVZ was typical for genes characteristic for the qNSCs state. Furthermore, we found many upregulated genes typical for astrocytes and oligodendrocytes, possibly related to the up-regulation of WNT and BMP signaling pathways (Han et al., 2014; Guo et al., 2015). Because qNSCs and astrocytes share many common gene expression profiles (Götz et al., 2015) the enrichment of astrocytic fate in the ischemic primate SVZ further support a prevalence of progenitors in a quiescent state.

Under steady-state conditions, the SVZa cells in the adult baboon monkey are enriched for the repressive chromatin marks H3K9me3 that point at a heterochromatin state (Foret et al., 2014). Ischemia in macaque brain induced expression of ∼1000 genes (15% of the total genes expressed in SVZa) which is likely to be accompanied by changes in the epigenetic state of the induced genes. It will be interesting to explore how ischemia affects the presence of repressive chromatin marks in monkey SVZa and which genes have retained or lost this mark. For example, the macaque transcriptome data did not reveal statistically significant expression changes of genes critical of NSC activation, including *EGFR* (Codega et al., 2014; Dulken et al., 2017) and *INFGR1* (Llorens-Bobadilla et al., 2015). It thus appears that more than a week is necessary to transform the transcriptional landscape of the macaque SVZa from a quiescent toward an active state, while in the rodent this transformation begins to take place on the second day following global ischemia (Llorens-Bobadilla et al., 2015). Another possible reason for the prevalence of qNSC-like gene signatures is an increase of the NOTCH and BMP signaling pathways detected at postischemic day 7, particularly in the EL. These two pathways are responsible for preserving quiescence of both NSCs (Chapouton et al., 2010; Llorens-Bobadilla et al., 2015) and ependymal cells (Carlén et al., 2009; Luo et al., 2015).

The postischemic response of brain stem cells has been studied in rodents (Napoli and Borlongan, 2016; Dillen et al., 2019; Nakagomi et al., 2019). While in the adult rodent brain, the ependymal cells are quiescent and express Notch signaling components, upon stroke they become activated to produce neuroblasts and glial cells. This requires Notch down regulation in the ependyma (Carlén et al., 2009). We find, in the macaque, seven-days after ischemia, an *enhanced* ependymal expression of members of the Notch signaling pathway (*Notch1, RBPJ, LFNG*). This upregulation of Notch signaling may be the reason for the inability of primates to initiate neurogenesis. A caveat is that down-regulation of Notch signaling could occur but after the 7 day time point.

### Genes With a Novel Expression Pattern in Primate SVZa

We report the expression pattern of 38 genes previously not documented in the mammalian SVZ. An intriguing example is the enzyme Stearoyl-CoA Desaturase 5. *SCD5* is not present in rodents (Wang et al., 2005). We found a strong postischemic activation of *SCD5* in the EL. Interestingly, SCD5 can inhibit cell proliferation by suppressing the EGFR signaling pathway (Sinner et al., 2012). This could contribute to the prevalence of qNSCs in the postischemic monkey SVZa. A strong postischemic enhancement in the EL was observed for the *Kinesin Family Member 2A* (*KIF2A*), a mitotic spindle regulator (Wordeman and Mitchison, 1995) involved in human cortical development (Cavallin et al., 2017). Another interesting example was the transcription factor *Myc-Associated Factor X* (*MAX*). The MAX protein forms heterodimers with several other transcription factors, most notably with c-MYC, which is critical in maintaining stemness of embryonic stem cells (Yoshida, 2018).

Two of the novel genes in the primate SVZa were shown to play a role in non-neuronal stem cell niches. These include *LGR5*, expressed in intestinal stem cells (Barker et al., 2007) and *ZBTB16*, which marks immature hematopoietic progenitors (Liu, 2007). Interestingly, we found a strong postischemic expression of both genes in EL and SEL. LGR5 is not expressed in the mouse SVZ (Yu et al., 2017) and its presence in the primate SVZa may indicate a primate-specific expression. During embryonic chick development, *ZBTB16* maintains the self-renewal of neural progenitors (Gaber et al., 2013), and its potential involvement in adult primate neurogenesis needs to be further studied.

### APLNR Exhibits a Strong Expression in Primate SVZa

Our data show, for the first time, expression of *APLNR* in the EL and SEL of the primate SVZa. This *APLNR* transcription is markedly increased upon ischemia. Studies reporting the transcriptomes of murine SVZ cell types (Beckervordersandforth et al., 2010; Codega et al., 2014; Llorens-Bobadilla et al., 2015; Dulken et al., 2017; Zywitza et al., 2018) did not detect *Aplnr* mRNA. We show here that such expression was very weak in the mouse SEL and undetectable in the EL, while in the control primate brain, both SEL and EL showed moderate presence of *APLNR* transcripts, implicating possible contribution to brain neurogenesis. Noteworthy, genetic *APLNR* variants have been associated with the increased stroke risk in humans (Hata et al., 2007). In rodents, activation of the apelinergic system improves the recovery after experimental stroke by inhibiting neuronal apoptosis and facilitating angiogenesis (Chen et al., 2015; Wu et al., 2017). Our data showing *APLNR* transcripts in *de novo* generated SVZa progenitors suggest that *APLNR* might contribute not only to postischemic neuroprotection, but also to regeneration after brain injury. Because APLNR is a druggable target, this receptor can serve as an entry point of clinically relevant molecules into a wide spectrum of primate SVZa cells.

## Data Availability Statement

The datasets presented in this study can be found in online repositories. The names of the repository/repositories and accession number(s) can be found in the article/Supplementary Material.

## Ethics Statement

The animal study was reviewed and approved by Animal Care and Ethics Committee of Kanazawa University Graduate School of Medical Sciences, Japan.

## Author Contributions

AS, GE, and AT designed the experiments. MC, CT, N-HH, IV, SP, and TY performed the experiments. HW, LW, and SP programed the data. MC, HW, SP, AS, GE, and AT analyzed the data. JY, GE, and AT supervised the project. AS, GE, and AT wrote the manuscript. All contributing authors commented on the manuscript and contributed to the article and approved the submitted version.

## Conflict of Interest

The authors declare that the research was conducted in the absence of any commercial or financial relationships that could be construed as a potential conflict of interest.

## Footnotes

1 www.monkey-niche.org

2 https://web.archive.org/web/20190414050927/http://www.rhesusbase.org/

3 https://david.ncifcrf.gov/

4 http://string-db.org/

5 https://cytoscape.org/

6 https://openslide.org/

7 https://openseadragon.github.io/

8 http://www.python.org/

9 http://www.pythonware.com/

10 https://github.com/biocompibens/SME/

11 https://github.com/StoyanPv/MKY-FISH_scripts

## Abbreviations

Bv: blood vessel
CC: corpus callosum
CG: cingulate gyrus
CSVZ: the callosal aspect of the subventricular zone
EL: ependymal layer
FrCx: frontal cortex
GO: Gene ontology
GSEA: gene set enrichment analysis
ISH: *in situ* hybridization
LV: lateral ventricle
NSC: neural stem cell
PVSEL: perivascular subependymal layer
SEL: subependymal layer on the striatal side
SPT: septum
STR: striatum
SVZa: subventricular zone along the anterior horn of LV
TAP: transit amplifying progenitor
Th: thalamus
aNSC: activated NSC
qNSC: quiescent NSC.
