# Supplementary figures and images for: Transcriptome Response and Spatial Pattern of Gene Expression in the Primate Subventricular Zone Neurogenic Niche After Cerebral Ischemia
Source: Front Cell Dev Biol. 2020 Dec 3;8:584314. doi: 10.3389/fcell.2020.584314 (PMC7744782; doi:10.3389/fcell.2020.584314)

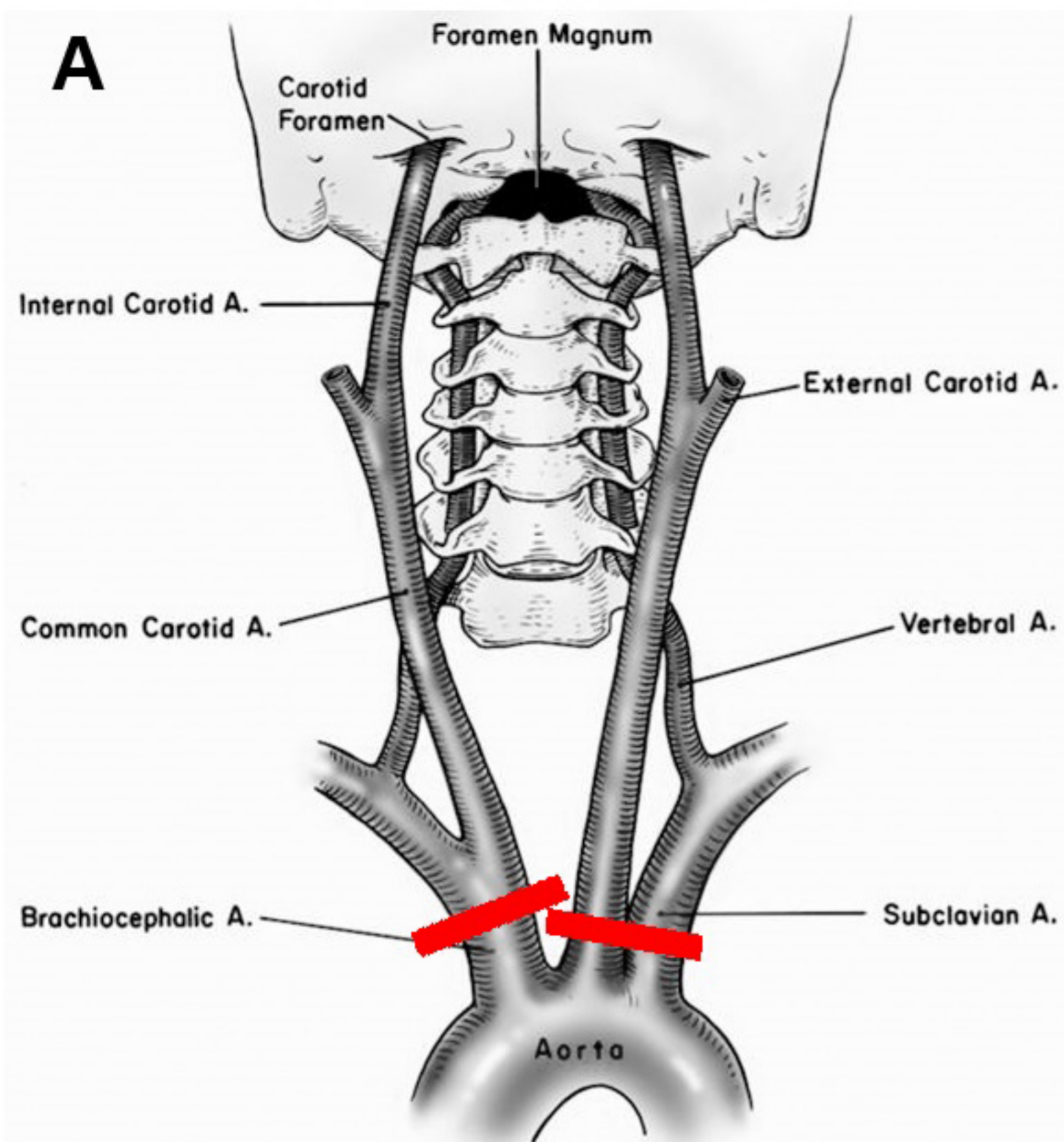

**B1** Coronal plane in B2

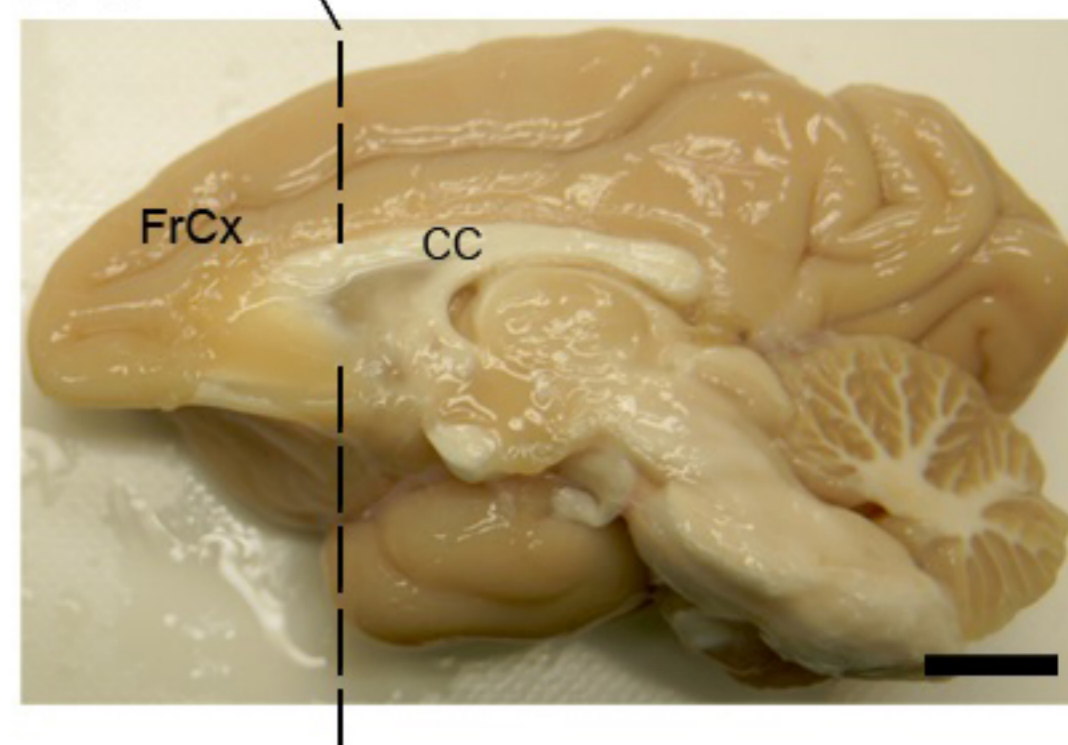

**B2**

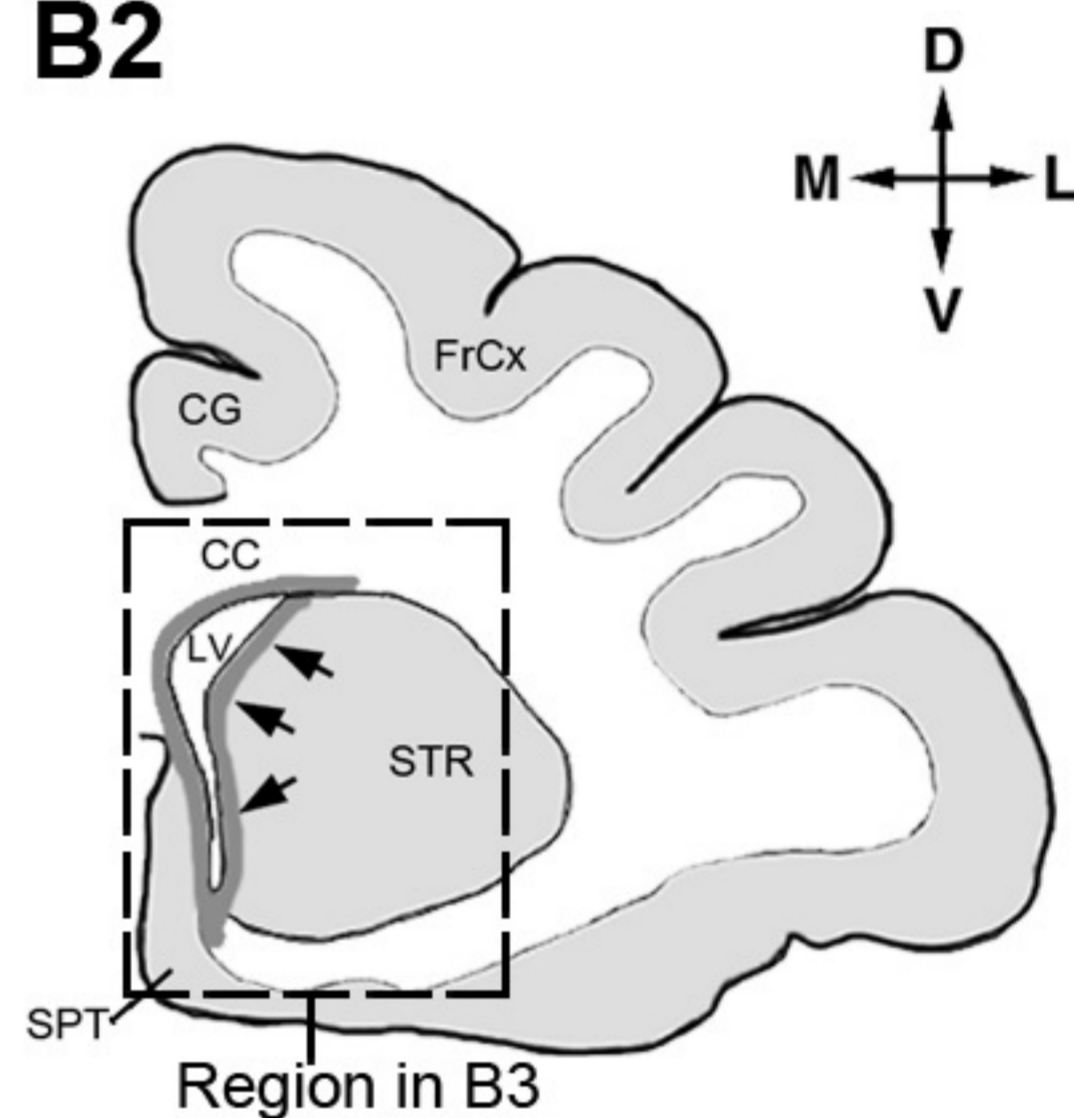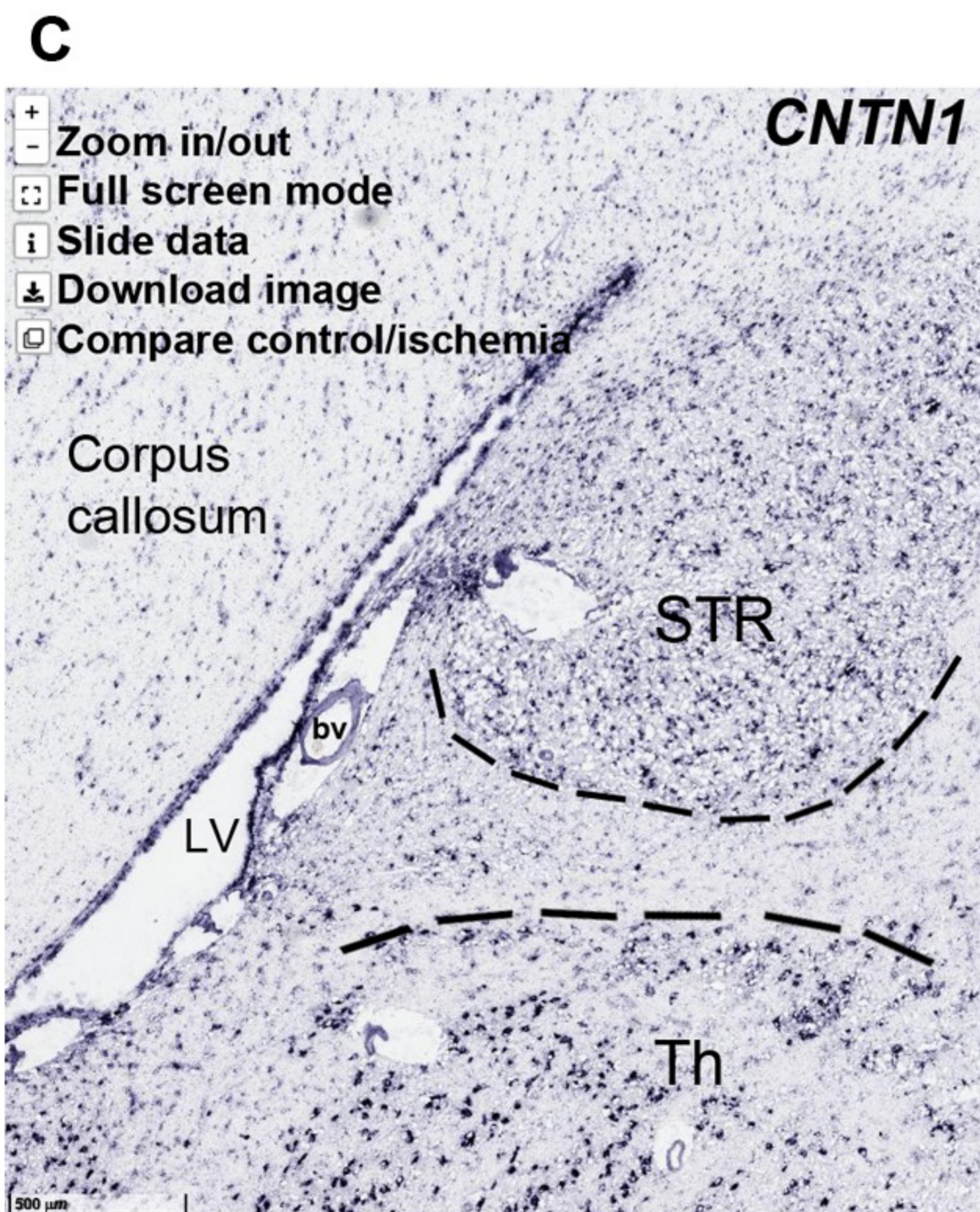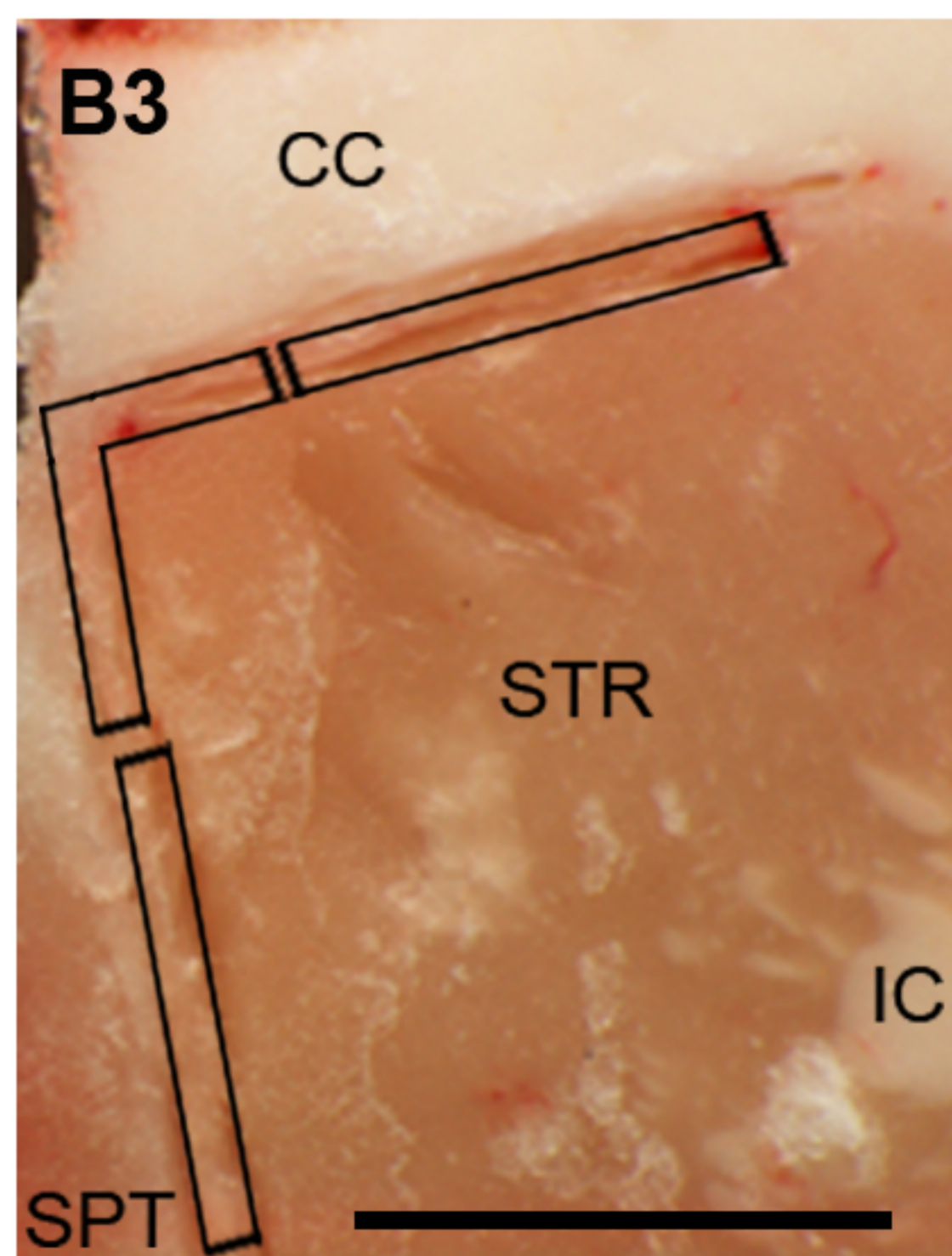

Supplement: Supplementary file 1 [file Image_1.pdf]

**A**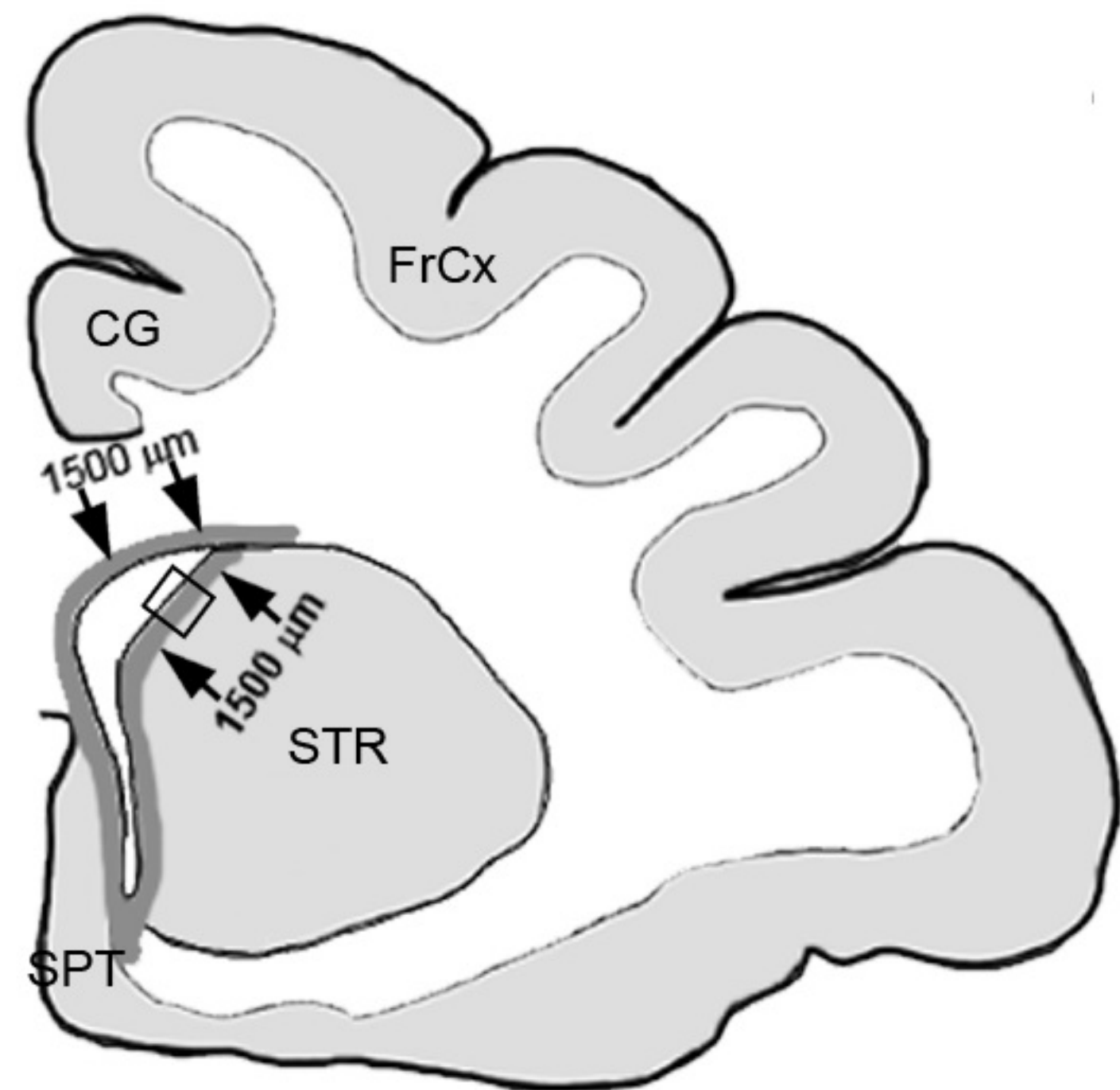**B**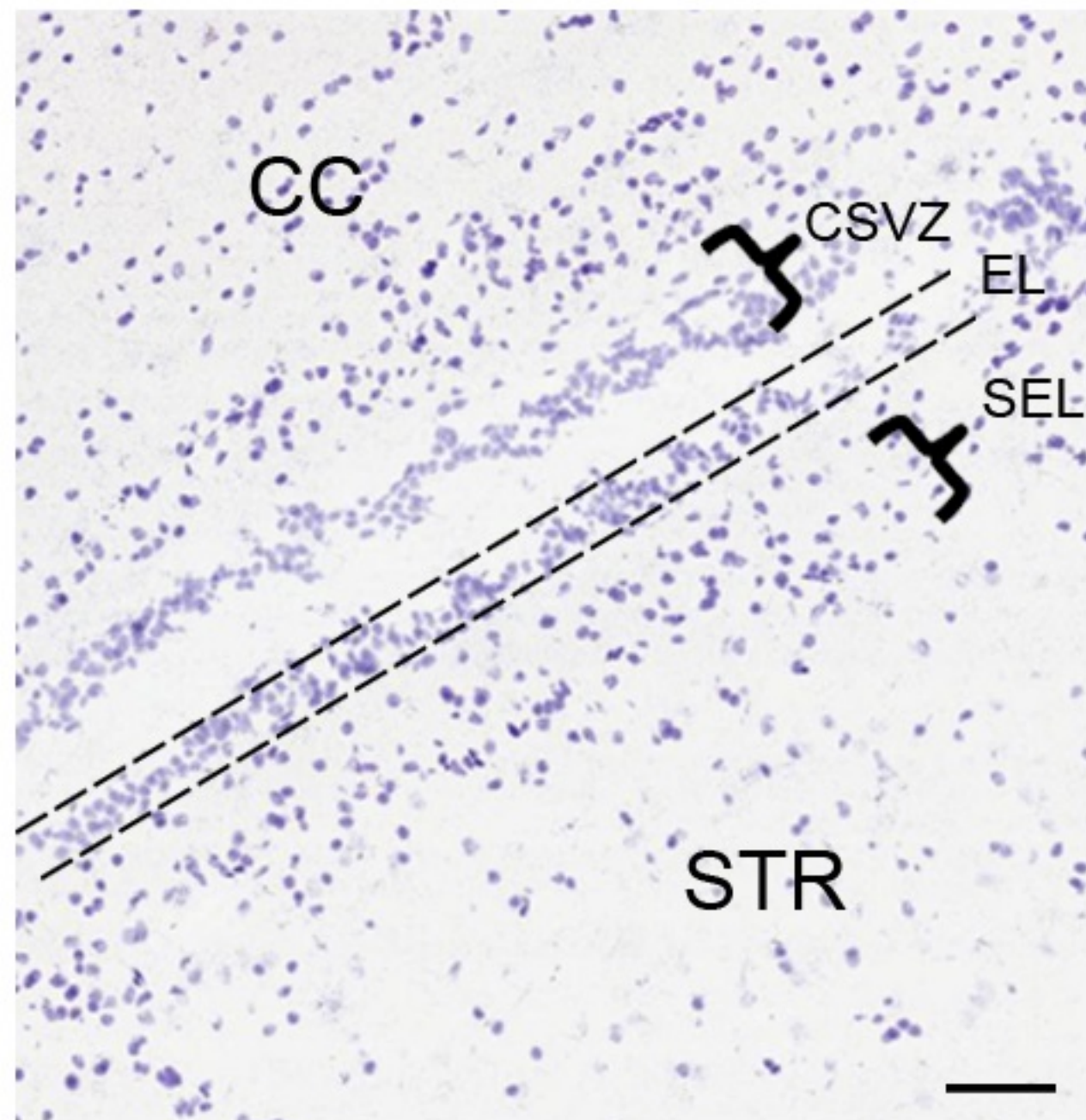

Supplement: Supplementary file 2 [file Image_2.pdf]

**A**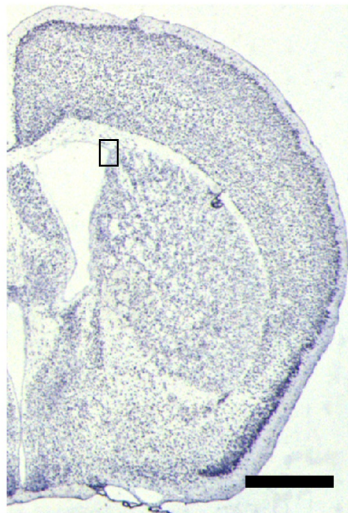**B**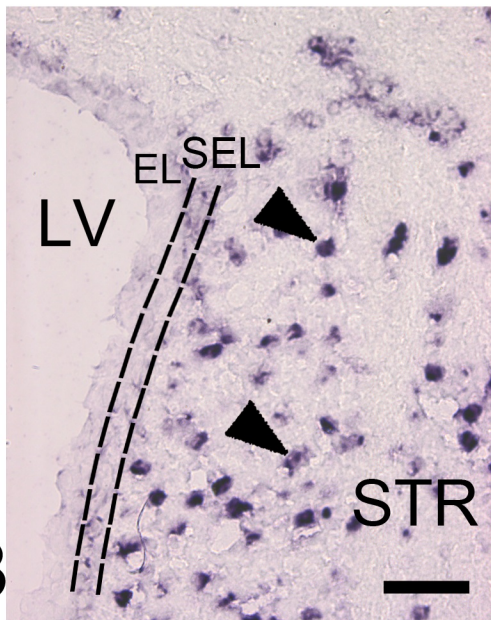

Supplement: Supplementary file 4 [file Image_4.pdf]
