# Supplementary material for: Transcriptome Response and Spatial Pattern of Gene Expression in the Primate Subventricular Zone Neurogenic Niche After Cerebral Ischemia
Source: Front Cell Dev Biol. 2020 Dec 3;8:584314. doi: 10.3389/fcell.2020.584314 (PMC7744782; doi:10.3389/fcell.2020.584314)
Supplement: Supplementary file 3 [file Image_3.pdf]

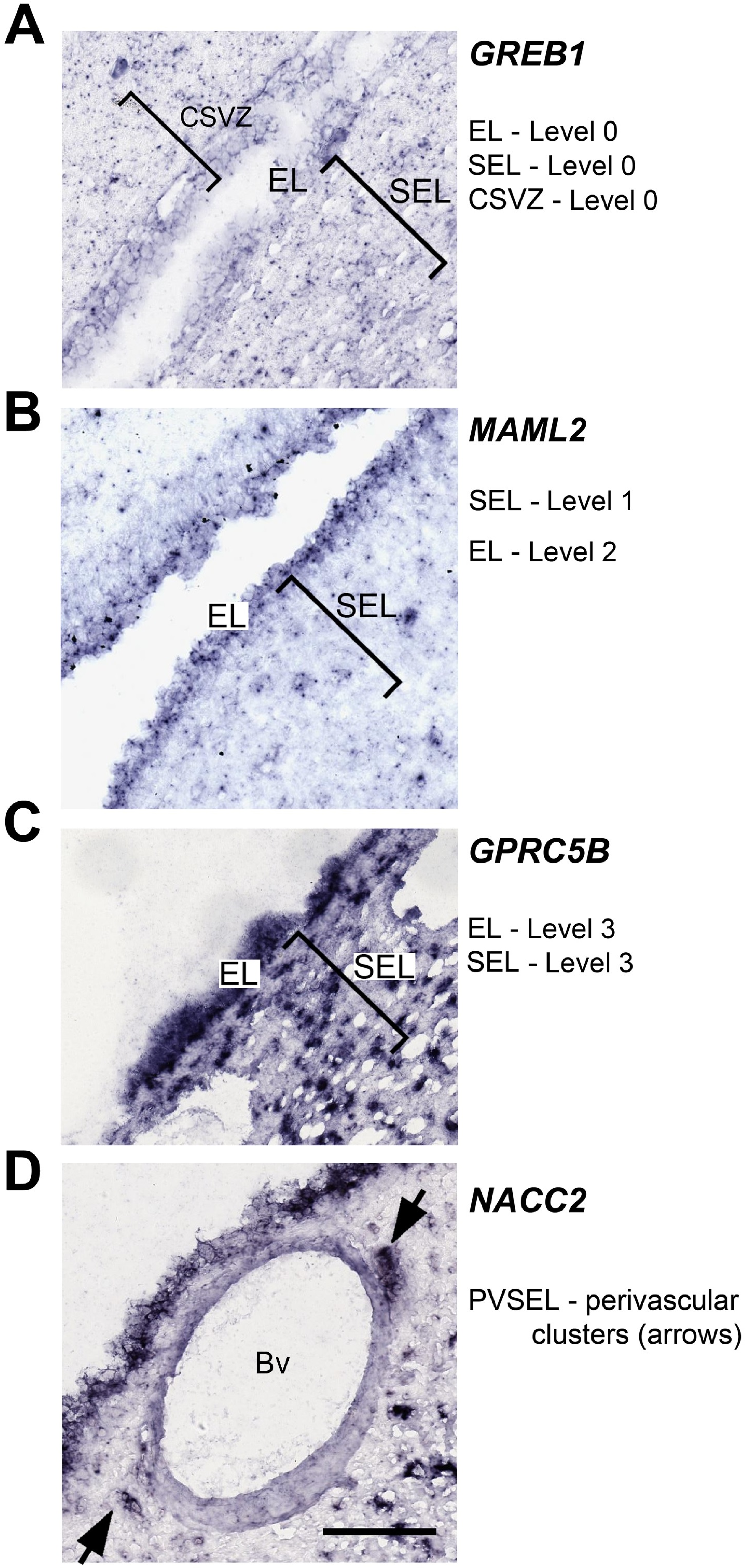
A
CSVZ
EL
SEL
GREB1
EL - Level 0
SEL - Level 0
CSVZ - Level 0
B
EL
SEL
MAML2
SEL - Level 1
EL - Level 2
C
EL
SEL
GPRC5B
EL - Level 3
SEL - Level 3
D
Bv
NACC2
PVSEL - perivascular
clusters (arrows)
