# Supplementary material for: Transcriptome Response and Spatial Pattern of Gene Expression in the Primate Subventricular Zone Neurogenic Niche After Cerebral Ischemia
Source: Front Cell Dev Biol. 2020 Dec 3;8:584314. doi: 10.3389/fcell.2020.584314 (PMC7744782; doi:10.3389/fcell.2020.584314)
Supplement: Supplementary file 5 [file Presentation_1.pdf]

Chongtham et al., Supplementary Legends

Supplementary Figure Legends

Supplementary Figure 1. **Anatomy of the monkey arterial tree and of primate SVZa.**

(**A**) Gross anatomy of the major arteries supplying the primate brain with the position of the clips (red transverse bars) used to temporarily block the cerebral blood flow to the macaque brain.

(**B**) Neuroanatomy of primate SVZa and adjacent brain regions. A medial view on the macaque brain right hemisphere (**B1**) at the level of the head of the caudate nucleus. (**B2**) A scheme of a coronal brain section (atlas level +25 mm relative to the interaural plane) shows the main brain regions adjacent to the SVZa, which is marked by dark gray shading (three arrows correspond to the three frames in **B3**). (**B3**) Photograph of a coronal slice corresponding to the area in a box in B2. Three frames correspond to the striatal periventricular tissue slabs which were dissected from each monkey brain and used for RNA sequencing.

(**C**) Screen shot from the "monkey-niche.org" database showing ISH staining of the *CNTN1* gene, and the interactive buttons used to navigate in the "monkey-niche" online atlas. For details see the online instructions.

Scale bars 1 cm (**B1**), 1 mm (**B3**), 500 μm (**C**).

Supplementary Figure 2. **Zones of quantitative evaluation of gene expression on tissue sections using Celldetekt.**

(**A**) A scheme of monkey brain as shown in Supplementary Figure B2. Arrows illustrate the length (1500 μm from the dorsal tip of the lateral ventricle) of the areas used for the analysis.

(**B**) Histological image (Cresyl violet staining) of the region outlined by a square in (**A**). The CSVZ, EL and SEL are outlined. Scale bar 100 μm.

Supplementary Figure 3. **Images illustrating the visual scoring of expression strength by an expert annotator in non-ischemic control tissue**

(**A**) Expression scores level 0 for *GREB1*.

(**B**) Expression of *MAML2* scores level 1 in the SEL and level 2 in EL.

(**C**) Expression of *GPRC5B* scores level 3 in EL and SEL.

(**D**) Expression of *NACC2* in perivascular clusters (PVSEL) scored as "present".

Scale bar 100 μm.

Supplementary Figure 4. **Colorimetric ISH for Aplnr in mouse SVZ niche.**

(A) Coronal section at low magnification showing an overview of the *Aplnr* signal. A box depicts the regions shown at high magnification in Panel B.

(**B**) A higher magnification micrographs in dorsal SVZ. A weak signal is present in the SEL (outlined by a dashed line) while the EL is not labeled. A strong *Aplnr* signal is evident in STR cells (arrowheads). Scale bars 1 mm (**A**), 20 μm (**B**).

Supplementary Table Legends

Supplementary Table 1.

Atlas coordinates of the sections shown in Figures 3-6.

Lists the atlas coordinates (according to Saleem and Logothetis, 2007) of the sections, images from which are presented in the ISH panels of Fig. 3D-F, Fig. 4D-K, Fig. 5, and 6C-F.

Supplementary Table 2, related to Figure 1A

All transcripts and their corresponding genes expressed in SVZa (total SVZa genes).

Lists of all transcripts and genes expressed in SVZa (average RPKM >0.5 in both control and ischemic monkeys). The average FPKM values for each transcript or gene in each of the 3 control and 3 ischemic monkeys are shown.

Supplementary Table 3, related to Figure 1A

Genes differentially expressed in SVZa after ischemia.

Lists of upregulated (SVZa-DE-UP) or down-regulated (SVZa-DE-DOWN) genes in SVZa 7 days after global cerebral ischemia.

Supplementary Table 4, related to Figure 2A,B

Gene Ontology categories for genes differentially expressed after ischemia.

Lists of genes differentially expressed in SVZa 7 days after global brain ischemia, grouped by GO categories.

Supplementary Table 5, related to Figure 2C

Gene set enrichment analysis (GSEA) of SVZa-DE genes.

Lists of enriched GSEA sets for the monkey SVZa-DE-UP and SVZa-DE-DOWN genes. Fisher's exact test was applied to identify the sets that show difference between SVZa-DE-UP *and* SVZa-DE-DOWN genes ("fisher.p" represents Fisher's test p-value).

Supplementary Table 6, related to Figure 2D

Transcription factors differentially expressed in SVZa after ischemia.

Lists of the transcription factors among the SVZa-DE-UP or SVZa-DE-DOWN genes, grouped by gene families.

Supplementary Table 7, related to Figure 1C.

List of the genes in the "monkey-niche" database.

Alphabetical lists the 150 genes either induced by ischemia or associated with stem cell biology, currently included in the interactive ISH atlas.

Supplementary Table 8, related to Figure 3A.

Comparison of monkey SVZa-DE genes with the cell type-enriched transcription profiles of Zhang et al., 2014.

Lists of SVZa-DE-UP or SVZa-DE-DOWN monkey genes present in the top 500 genes (downloaded from http://www.brainrnaseq.org/) characteristic for the following adult mouse cortex cell types: astrocytes, oligodendrocyte precursor cells, newly formed

oligodendrocytes, myelinating oligodendrocytes, microglial cells, endothelial cells, and neurons. The Fisher's test p-value and the odd ratio are reported for each cell type.

Supplementary Table 9, related to Figure 4A.

Comparison of monkey SVZa-DE genes with the data of Codega et al., 2014.

Lists of the SVZa-DE-UP and SVZa-DE-DOWN monkey genes present in the datasets of Table S1 of Codega et al., 2014. The Fisher's test p-value and the odd ratio are reported for each cell type.

Supplementary Table 10, related to Figure 4A.

Comparison of monkey SVZa-DE genes with the data of Beckervordersandforth et al., 2010.

Lists of the SVZa-DE-UP and SVZa-DE-DOWN monkey genes present in Tables S2, S3, and S5 of Beckervordersandforth et al., 2010. The Fisher's test p-value and the odd ratio are reported for each cell type.

Supplementary Table 11, related to Figure 4A.

Comparison of monkey SVZa-DE genes with the data of Dulken et al., 2017.

Lists of the SVZa-DE-UP and SVZa-DE-DOWN monkey genes present in the datasets of Dulken et al., 2017. To determine the genes enriched in SVZ progenitor populations, the normalized expression data were downloaded from NCBI BioProject: PRJNA324289. For each gene of the dataset of Dulken et al., 2017 we calculated the mean expression value in each cell type defined in their study. The genes with mean expression value>10 and fold change >3 were compared to the monkey SVZa-DE genes. The Fisher's test p-value and the odd ratio are reported for each cell type.

Supplementary Table 12, related to Figure 4A.

Comparison of monkey SVZa-DE genes with the data of Llorens-Bobadilla et al., 2015.

Lists of the SVZa-DE-UP and SVZa-DE-DOWN monkey genes present in Table S2 of Llorens-Bobadilla et al., 2015. The Fisher's test p-value and the odd ratio are reported for each cell type.

Supplementary Table 13, related to Figure 5.

Genes enhanced by ischemia which belong to NOTCH, BMP or WNT signaling pathways.

Lists of the SVZa-DE-UP genes which belong to major signaling pathways as defined by applying GSEA. The MAPK / signaling pathway-related genes were extracted from the KEGG database (https://www.genome.jp/kegg/pathway.html). The WNT signaling pathway-related genes were extracted from the Broad Institute database (http://software.broadinstitute.org/gsea/msigdb/cards/wnt_signaling.html). The BMP signaling pathway-related genes were extracted from the Pathway Interaction Database (PID, http://www.ndexbio.org/). The NOTCH signaling pathway-related genes were extracted from the from REACTOME database (https://reactome.org/). FDR, false discovery rate (GSEA). The SVZa-DE-DOWN genes were not enriched in NOTCH, BMP or WNT signaling pathways.

Supplementary Table 14, related to Figure 7.

Analysis of cell identity in monkey SEL.

This table reports the number of control monkeys from which sections are stained by fluorescent ISH or immunostaning, the number of brain sections analyzed in semi-automated manner for each molecule, the number of DAPI+ nuclei segmented during the analysis, and the absolute number of cells found positive for each molecule.
